# Activation of the Pseudocapacitive Behavior of MXene/PANI for High‐Performance Ammonium‐Ion Batteries

**DOI:** 10.1002/advs.202511815

**Published:** 2025-08-21

**Authors:** Yeying Li, Leixi Du, Liping Zhang, Changjie Huang, Justinas Palisaitis, Jingkun Xu, Johanna Rosen, Jianxia Jiang, Leiqiang Qin

**Affiliations:** ^1^ Flexible Electronics Innovation Institute (FEII) Jiangxi Provincial Key Laboratory of Flexible Electronics Jiangxi Science and Technology Normal University Nanchang 330013 China; ^2^ School of Chemistry and Chemical Engineering Jiangxi Science and Technology Normal University Nanchang Jiangxi 330013 China; ^3^ Department of Physics Chemistry and Biology (IFM) Linköping University Linköping 58183 Sweden

**Keywords:** aqueous ammonium ion battery, MXene, non‐metallic ions, proton enhancement, pseudocapacitive behavior

## Abstract

The development of aqueous ammonium‐ion batteries (AAIBs) requires electrode materials that combine high NH_4_^+^ storage capacity with rapid and reversible ion transport. Herein, a metal‐vacancy MXene/polyaniline (Mo_4/3_CT_z_/PANI) composite is reported, in which the pseudocapacitive response is synergistically activated by introducing 0.1 m H_2_SO_4_ into 1 m (NH_4_)_2_SO_4_ electrolyte. This proton‐assisted modulation enables rapid and reversible NH_4_^+^/H_3_O^+^ co‐intercalation, in contrast to the negligible ion insertion observed in the absence of H_2_SO_4_. Combined experimental and density‐functional theory (DFT) analyses reveal that proton doping significantly improves the electronic conductivity of PANI and induces a reversible Mo^6+^/Mo^5+^ redox transition during cycling, which dynamically modulates the NH_4_^+^ adsorption energy (from −4.155 to −4.567 eV), thus facilitating both intercalation and deintercalation of NH_4_^+^. As a result, the composite achieves a high specific capacity of 245 mAh g^−1^ at 0.1 A g^−1^, with excellent capacity retention of 84.2% after 11,000 cycles at 1.0 A g^−1^. Furthermore, the MnO_2_/CNTs||M:P = 5:1 full cell delivers a high energy density of 81.6 Wh kg^−1^ and a power density of 16 000 W kg^−1^. This work highlights a promising strategy for advancing MXene‐based electrodes via proton‐enhanced ion storage mechanisms, paving the way for high‐performance AAIBs.

## Graphical abstract

A Mo_4/3_CT_z_/PANI composite with in situ grown PANI nanosheets forming a wrinkled structure is developed to stabilize interlayer spacing and enhance charge transport. Proton addition via H_2_SO_4_ activates pseudocapacitive behavior in both components by modulating Mo valence and improving PANI conductivity. This synergistic modulation lowers the NH_4_^+^ adsorption energy barrier and promotes its intercalation, thereby enabling efficient NH_4_^+^/H_3_O^+^ co‐storage.

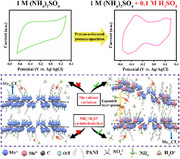

## 1 Introduction

Rechargeable aqueous batteries have emerged as a promising research direction in the field of energy storage due to their inherent safety, environmental friendliness, and low cost, making them well‐aligned with the principles of sustainable development and promising for large‐scale applications.^[^ ^1^, ^2^, ^3^ ^]^ Various types of aqueous ion batteries and hybrid supercapacitors have been proposed and extensively investigated. However, the development of metal‐ion‐based aqueous energy storage systems is constrained by several challenges, such as the complex side reactions at the electrode–electrolyte interface^[^ ^4^ ^]^ and significant volume expansion of cathode materials during metal ion intercalation, particularly in systems involving multivalent metal ions, thereby compromising long‐term structural stability.^[^ ^5^ ^]^ Recently, non‐metallic ions have emerged as alternative charge carriers, exhibiting pseudocapacitive behavior comparable to that of metal ions.^[^ ^6^, ^7^ ^]^ This shift offers new opportunities for designing cost‐effective, stable, and environmentally friendly aqueous energy storage systems. Among them, ammonium ions (NH_4_^+^) are particularly attractive due to their smaller hydrated ionic radius (3.31 Å), lower molar mass (18 g mol^−1^), and rapid diffusion capabilities in aqueous electrolytes.^[^ ^8^, ^9^, ^10^ ^]^ Moreover, NH_4_^+^ circumvents dendrite formation and electrochemical plating issues commonly observed in metal‐ion systems, significantly reducing safety risks. Specifically, NH_4_^+^ can form hydrogen bonds with host materials,^[^ ^11^, ^12^, ^13^ ^]^ which enhances both the capacity and structural stability of the electrode. Therefore, the design and engineering of electrode materials for high‐performance aqueous ammonium‐ion batteries (AAIBs) hold considerable potential for next‐generation sustainable energy storage.

In recent years, various electrode materials have been explored for NH_4_^+^ storage in aqueous electrolytes, such as Prussian blue analogues,^[^ ^14^ ^]^ transition metal oxides/sulfides,^[^ ^15^ ^]^ and organic polymers.^[^ ^16^ ^]^ However, these systems often suffer from low electronic conductivity and sluggish redox reaction kinetics, which hinder their practical performance. MXenes, a class of emerging two‐dimensional materials,^[^ ^17^, ^18^ ^]^ have attracted considerable attention due to their exceptional electrical conductivity, fast pseudocapacitive behavior, and adjustable interlayer spacing,^[^ ^19^, ^20^, ^21^ ^]^ making them promising candidates for not only aqueous metal‐ion batteries but also for NH_4_^+^ storage.^[^ ^22^ ^]^ Nevertheless, the tetrahedral geometry of NH_4_^+^ and its strong hydrogen‐bonding interactions with host frameworks introduce additional complexity into the diffusion process, which involves continuous bond breaking and reformation along preferential pathways.^[^ ^23^ ^]^ This unique behavior places stringent demands on the host material, which must simultaneously i) provide abundant, energetically favorable NH_4_^+^ adsorption sites to enable highly reversible ion intercalation and ii) possess sufficiently large interlayer spacing to facilitate fast NH_4_^+^ transport. Therefore, rational engineering of the morphological, chemical, and electronic structures of MXenes is crucial for the development of high‐performance AAIBs.

Recent advances in emerging material systems have created new opportunities for enhancing NH_4_^+^ storage performance in MXene‐based hosts. Among them, the first in‐plane vacancy‐ordered MXene (Mo_4/3_CT_z_), reported in 2017,^[^ ^18^ ^]^ features periodically distributed metal vacancies that expose a higher density of electrochemically active sites. Compared to its vacancy‐free counterpart Mo_2_C, Mo_4/3_CT_z_ exhibits significantly improved pseudocapacitive behavior and outstanding structural stability in 1 m H_2_SO_4_ electrolyte, demonstrating strong proton storage capability. Meanwhile, polyaniline (PANI), a prototypical conductive polymer^[^ ^24^, ^25^ ^]^ and widely studied NH_4_^+^ host,^[^ ^16^, ^26^ ^]^ offers high pseudocapacitance due to its intrinsic redox activity and doping chemistry in acidic environments,^[^ ^24^, ^27^ ^]^ making it an ideal candidate for composite construction. Considering the strong proton affinity of both Mo_4/3_CT_z_ and PANI, along with the structural and functional versatility of protons and NH_4_^+^ ions, their co‐coordination within composites is expected to overcome kinetic and stability limitations, enabling rapid and durable energy storage.

Building on these insights, we developed a proton‐regulated Mo_4/3_CT_z_ MXene/PANI composite to enable efficient high ammonium‐ion storage. Through compositional optimization, PANI nanosheets grow in situ along the MXene surface to form a wrinkled structure, which serves as a structural pillar to stabilize the interlayer spacing and facilitate efficient charge and ion transport. Proton introduction, achieved by adding 0.1 m H_2_SO_4_ to 1 m (NH_4_)_2_SO_4_ electrolyte, not only improves the electronic conductivity of PANI but also induces a reversible Mo^6+^/Mo^5+^ redox transition during cycling. Density‐functional‐theory (DFT) calculations reveal that the Mo^6+^/Mo^5+^ valence transition dynamically alters the NH_4_^+^ adsorption energy from −4.155 eV (Mo^6+^) to −4.567 eV (Mo^5+^) during cycling, thereby facilitating both NH_4_^+^ intercalation and deintercalation. Furthermore, the MXene‐polymer interface provides favorable adsorption sites, which facilitate charge redistribution and local electric field regulation following NH_4_^+^ adsorption, thereby further promoting the intercalation process. As a result, the Mo_4/3_CT_z_/PANI composite delivers a high specific capacity of 245 mA h g^−1^ at 0.1 A g^−1^ in a mixed electrolyte of 0.1 m H_2_SO_4_ and 1 m (NH_4_)_2_SO_4_, maintaining 84.2% capacity retention after 11 000 cycles. Furthermore, the assembled MnO_2_/CNT||M:P = 5:1 full cell demonstrates an energy density of 81.6 Wh kg^−1^ at a power density of 2985 W kg^−1^, and exhibits excellent cycling stability, retaining 75.5% of its capacity after 10 000 cycles at 1 A g^−1^. These findings highlight the effectiveness of proton‐activated and interfacial engineering strategies in addressing both kinetic and stability challenges in AAIBs.

## 2 Results and Discussion

**Figure 1:**
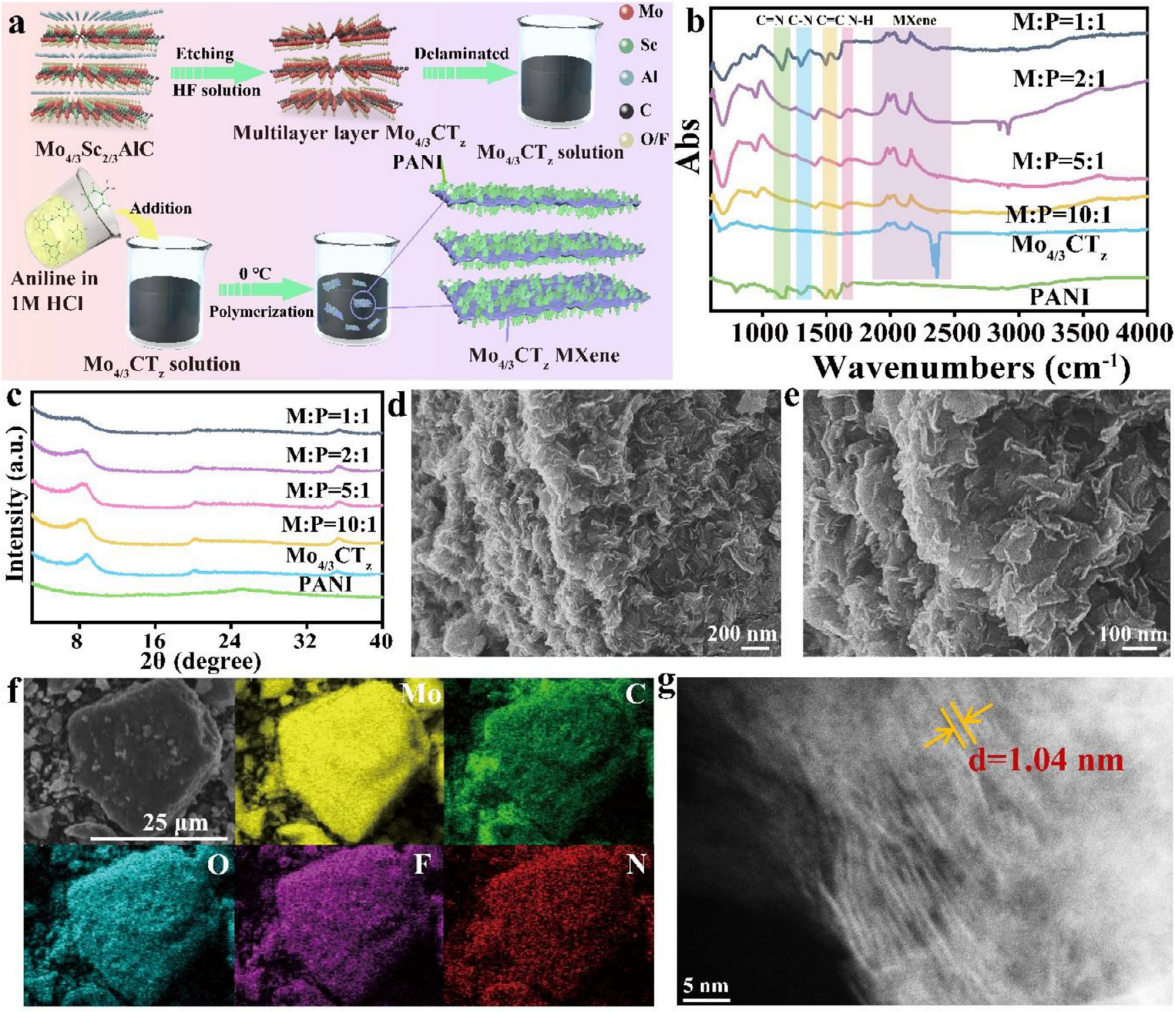
Structural characterization of Mo_4/3_CT_z_/PANI composite. a) Schematic illustration of the synthesis process of Mo_4/3_CT_z_/PANI composites. b) ATR‐FTIR spectra of Mo_4/3_CT_z_, PANI, and Mo_4/3_CT_z_/PANI composites with different ratios. c) X‐ray diffraction (XRD) patterns of Mo_4/3_CT_z_, PANI, and Mo_4/3_CT_z_/PANI composites with various mass ratios. d,e) Scanning electron microscopy (SEM) images of the M:P = 5:1 composite. f) Energy‐dispersive X‐ray spectroscopy (EDS) elemental mapping of Mo, C, O, F, and N for the M:P = 5:1 composite. g) STEM image of the M:P = 5:1 composite.

To achieve a high‐quality Mo_4/3_CT_z_/PANI composite, a series of composites with varying mass ratios of Mo_4/3_CT_z_ to PANI were prepared. The overall synthesis procedures are schematically illustrated in **Figure** **1**a. Specifically, an aqueous suspension of delaminated Mo_4/3_CT_z_ flakes was first prepared by selectively removing aluminum (Al) and scandium (Sc) from the Mo_4/3_Sc_2/3_AlC *i*‐MAX phase (Figure S1, Supporting Information). Subsequently, PANI was deposited onto the resulting MXene flakes through a low‐temperature in situ polymerization method. By precisely adjusting the feed ratio of Mo_4/3_CT_z_ to PANI, a series of composites with distinct mass compositions was successfully fabricated. Fourier transform infrared (FTIR) spectroscopy was employed to investigate the structural features of Mo_4/3_CT_z_, PANI, and their composites. As illustrated in Figure 1b, the Mo_4/3_CT_z_/PANI composites all exhibit the characteristic peaks of PANI, including C═N stretching vibration at 1167 cm^−1^, C─N stretching vibration at 1300 cm^−1^, C═C aromatic ring vibration at 1497 and 1584 cm^−1^, and N‐H bending vibrations at 1670 cm^−1^, as well as the presence of characteristic absorption bands corresponding to Mo_4/3_CT_z_ MXene, confirming the successful combination of the two components. X‐ray diffraction (XRD) analysis was conducted to examine the layered structure of the materials. As shown in Figure 1c, the (002) diffraction peak of Mo_4/3_CT_z_ shifts to a lower angle after being composited with PANI, indicating an enlarged interlayer spacing due to polymer intercalation.^[^ ^18^, ^25^, ^26^ ^]^ This expansion is expected to provide structural support at the interlayer level and potentially enhance the overall stability of the composite. Morphological investigations were conducted via scanning electron microscopy (SEM). As shown in Figure S2 (Supporting Information), the pristine Mo_4/3_CT_z_ exhibits a well‐stacked lamellar morphology, while PANI displays an irregular granular texture. The composite exhibits a heterogeneous layered structure, with PANI uniformly coating the Mo_4/3_CT_z_ surface. Notably, at a Mo_4/3_CT_z_‐to‐PANI mass ratio of 5:1 (denoted M:P = 5:1), PANI forms nanosheet‐like structures oriented roughly perpendicular to the MXene surface, resulting in a wrinkled morphology (Figure 1d,e; Figure S3, Supporting Information), distinct from the disordered structures at other ratios. This architecture alleviates the shielding of MXene terminal groups caused by dense PANI aggregation and simultaneously promotes the formation of efficient ion/electron transport channels on the composite surface. Furthermore, the SEM‐based energy dispersive X‐ray spectroscopy (EDS) elemental mapping (Figure 1f) confirmed the homogeneous distribution of Mo, C, O, N, and F elements throughout the M:P = 5:1 composite, supporting the uniform integration of PANI into the MXene matrix. This was further supported by scanning transmission electron microscopy (STEM) images (Figure 1g; Figure S4, Supporting Information), which clearly show a well‐defined layered interface, along with PANI distributed both across the MXene surface and within its interlayer spaces. A slight expansion of the interlayer spacing to ≈1 nm was observed, likely due to the intercalation of PANI.

To determine the optimal composition of the Mo_4/3_CT_z_/PANI composite, a series of films with varying Mo_4/3_CT_z_‐to‐PANI mass ratios was fabricated, along with pristine Mo_4/3_CT_z_ MXene and PANI films as references. The electrochemical performance of all samples was systematically evaluated in a three‐electrode system using a conventional 1 m (NH_4_)_2_SO_4_ electrolyte. Detailed electrode preparation procedures are provided in the Supporting Information. As shown in Figure S5a,b (Supporting Information), cyclic voltammetry (CV) curves recorded at a scan rate of 10 mV s^−1^ reveal that all composite samples exhibit profiles similar to that of pristine Mo_4/3_CT_z_. Notably, the composite with a mass ratio of M:P = 5:1 displays the largest integrated area under the CV curve, corresponding to the highest specific capacity of 52.5 mAh g^−1^ at a current density of 0.1 A g^−1^ (Figure S5d, Supporting Information). To further evaluate charge transfer kinetics, electrochemical impedance spectroscopy (EIS) was performed under the same electrolyte conditions. The resulting Nyquist plots and corresponding equivalent circuit model are presented in Figure S5c,f, (Supporting Information), respectively. The equivalent circuit consists of equivalent series resistance (R_s_), charge transfer resistance (R_ct_), and Warburg impedance (Z_w_). As summarized in Table S1 (Supporting Information), the M:P = 5:1 composite exhibits the lowest R_ct_ value (47.43 Ω) among all samples, indicating more efficient charge transfer and superior rate capability. Additionally, this sample shows the steepest slope in the low frequency region, suggesting reduced ion transport resistance and improved ion diffusion within the electrode. These observations suggest that composite engineering can accelerate electrolyte‐ion diffusion and improve overall electrochemical performance. This enhancement aligns with the characteristic wrinkled morphology formed by nanosheet‐like PANI structures in the M:P = 5:1 composite. Therefore, unless otherwise specified, the M:P = 5:1 composition is considered optimal and will be used for all subsequent analyses. However, it is noteworthy that the CV curves of all composites lack distinct reversible redox peaks, and the specific capacity related to NH_4_^+^ storage remains relatively low. This indicates that the energy storage potential of the materials has not yet been fully realized.

**Figure 2:**
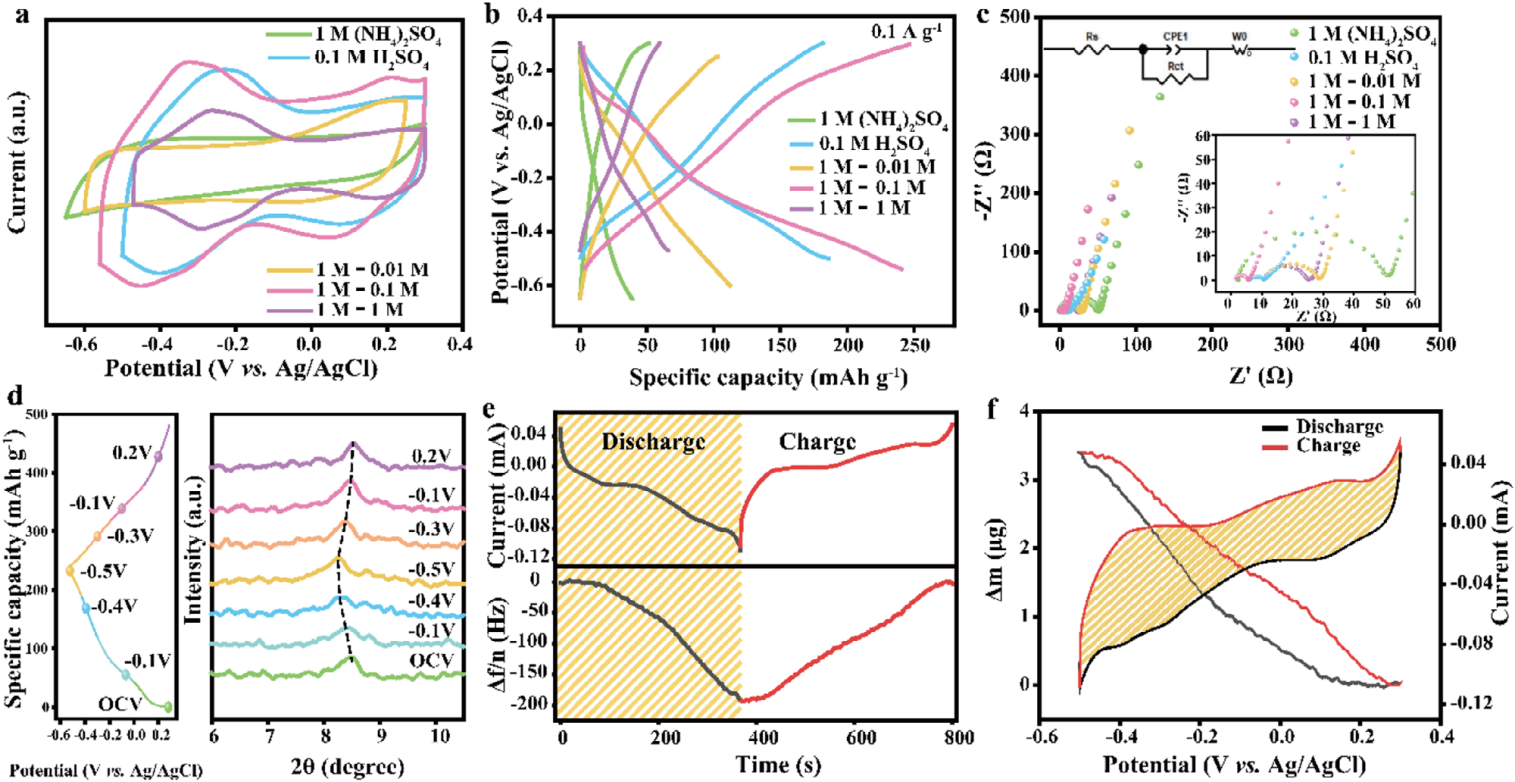
Electrolyte‐dependent electrochemical performance and charge storage behavior of the M:P= 5:1 composite. a) Cyclic voltammetry (CV) curves at a scan rate of 10 mV s^−1^ in different electrolytes. b) Galvanostatic charge‐discharge (GCD) curve at 0.1 A g^−1^ in different electrolytes. c) Electrochemical impedance spectroscopy (EIS) in different electrolytes. d) Ex situ XRD patterns and corresponding GCD curves at 0.1 A g^−1^ in 1–0.1 m electrolyte. e) Time‐resolved CV current (top) and the corresponding frequency shift (Δf/n, bottom) recorded for thin electrodes at a scan rate of 10 mV s^−1^ in 1–0.1 m electrolyte. f) CV profile and mass variation recorded during a single charge‐discharge cycle at 10 mV s^−1^ in the EQCM cell using a 1–0.1 m mixed electrolyte.

As a critical component of energy storage devices, the electrolyte plays a crucial role in determining overall performance. Leveraging the intrinsic redox activity of MXene in H_2_SO_4_ and the protonation‐enhanced conductivity of PANI, we systematically investigated the influence of H_2_SO_4_ as an additive on the charge‐storage behavior of the M:P = 5:1 composite. As illustrated in **Figure** **2**a,b, the electrochemical performance of the composite in various electrolyte systems was evaluated using cyclic voltammetry (CV) and galvanostatic charge‐discharge (GCD) measurements, respectively. In both 1 m (NH_4_)_2_SO_4_ and 1 m (NH_4_)_2_SO_4_ + 0.01 m H_2_SO_4_ electrolytes, the CV curves exhibit no apparent redox peaks. In contrast, when the concentration of H_2_SO_4_ was increased to 0.1 m or 1 m or when using pure 0.1 m H_2_SO_4_ two well‐defined pairs of redox peaks emerged, corresponding to the dual discharge plateaus observed in the GCD profiles (Figure 2b). These results highlight that electrolyte optimization can effectively activate redox processes in the composite. Although higher concentrations of H_2_SO_4_ narrow the electrochemical stability window by lowering the water decomposition potential, the mixed electrolyte comprising 1 m (NH_4_)_2_SO_4_ and 0.1 m H_2_SO_4_ (hereafter referred to as 1–0.1 m) maintains a broader operating voltage than pure 0.1 m H_2_SO_4_. Among all tested electrolytes, the 1–0.1 m mixed electrolyte delivers the highest charge storage capacity, as evidenced by the CV results in Figure 2a. These findings underscore the pivotal role of H_2_SO_4_ in regulating redox activity and enhancing the electrochemical performance of the composite electrode. To further evaluate the ion and charge transport properties of the composite in different electrolyte conditions, EIS was conducted on the M:P = 5:1 composite at open circuit potential (Figure 2c). The corresponding internal resistance (R_s_) and charge transfer resistance (R_ct_) were extracted from the Nyquist plots and are summarized in Table S2 (Supporting Information). The R_s_ value in the 1–0.1 m electrolyte (1.72 Ω) is significantly lower than those in the other electrolytes, indicating superior ionic conductivity, likely due to an optimal proton concentration that promotes hydrogen bonding with NH_4_^+^ ions and facilitates their transport. Moreover, the R_ct_ value in the 1–0.1 m electrolyte is also notably reduced (3.95 Ω), indicating enhanced electronic conductivity. The near‐vertical slope of the EIS curve in the low‐frequency region further suggests reduced ion‐diffusion resistance. Furthermore, we conducted additional electrochemical tests on M:P = 5:1 composite in sulfuric acid solutions of varying concentrations (Figure S6, Supporting Information). The results indicate that as the H_2_SO_4_ concentration increases from 0.01 to 0.1 m, the redox peak of M:P = 5:1 becomes more pronounced, the electrochemical capacity increases, and the impedance decreases. However, when the H_2_SO_4_ concentration is increased to 1 m, the intensity of the redox peak diminishes, and the capacity decreases significantly. This indicates that low proton concentrations can promote the capacitance performance of M:P = 5:1, but when the proton concentration is too high, it will reduce the capacitance. Together, these results confirm that the optimized mixed electrolyte greatly enhances NH_4_^+^ ion diffusion kinetics within the composite electrode.

To elucidate the mechanism by which H_2_SO_4_ enhances the electrochemical performance of the M:P = 5:1 composite, a series of spectroscopic and electrochemical characterizations were conducted to monitor the structural evolution during charge/discharge processes in various electrolytes. As shown in Figure 2d and Figure S7 (Supporting Information), ex situ XRD patterns of the M:P = 5:1 electrode were collected at selected charge/discharge states in 1–0.1 m, 0.1 m H_2_SO_4_, and 1 m (NH_4_)_2_SO_4_ electrolytes. In the mixed electrolyte (Figure 2d), the (002) diffraction peak shifts from 8.52° to 8.26° during discharge, indicating interlayer expansion due to ion intercalation. Upon charging, the peak reverts to its original position, reflecting reversible lattice contraction and confirming the high reversibility of ion intercalation/deintercalation in the mixed electrolyte.^[^ ^17^, ^39^, ^40^, ^41^ ^]^ In contrast, no significant peak shift is observed in 1 m (NH_4_)_2_SO_4_ (8.58°) or 0.1 m H_2_SO_4_ (8.86°), suggesting minimal or no effective intercalation. These results reveal clear electrolyte‐dependent ion intercalation behavior.

To identify the intercalating species responsible for the observed lattice expansion, in situ electrochemical quartz crystal microbalance (EQCM) measurements were performed in the mixed electrolyte system.^[^ ^28^ ^]^ As shown in Figure 2e, the M:P = 5:1 composite exhibited highly symmetric and fully reversible frequency shifts during charge‐discharge cycles, indicating a highly reversible mass‐change process. According to the Sauerbrey equation and Faraday's law, the apparent molar mass (M_v_, g·mol^−1^·e^−1^) of the participating species is related to the mass change per coulomb (Δm/ΔQ), which can be calculated from the slope in Figure 2f. In this study, the calculated M_v_ in the 1–0.1 M mixed electrolyte was ≈18.3 g·mol^−1^·e^−1^. Given that protons commonly contribute to charge storage in aqueous electrolytes, and that the theoretical molar masses of NH_4_^+^ and H_3_O^+^ are 18 and 19 g·mol^−1^, respectively, this intermediate value indicates that both ions contribute to the mass change during charge/discharge, thereby confirming a co‐intercalation mechanism. This conclusion is further corroborated by the consistency of this result with observed redox features (Figure 2a) and interlayer expansion behavior (Figure 2d). To further clarify the influence of electrolyte composition on intercalation kinetics, EQCM measurements were also performed in 1 m (NH_4_)_2_SO_4_ electrolyte as a control. As shown in Figure S8 (Supporting Information), the time‐dependent frequency curve in 1 m (NH_4_)_2_SO_4_ displays pronounced plateaus at the end of charging and the onset of discharging, indicating sluggish ion adsorption/desorption kinetics likely associated with a distinct energy barrier. In contrast, the continuous and smooth frequency shift observed in the mixed electrolyte suggests that protons lower the energy barrier for ion adsorption, thereby facilitating the intercalation process. This finding highlights the critical role of protons in modulating interfacial kinetics and supports the synergistic NH_4_^+^/H_3_O^+^ co‐intercalation mechanism. In addition, ex situ Raman spectroscopy (Figure S9, Supporting Information) provides further evidence for reversible ion intercalation in the mixed electrolyte. The characteristic N─H stretching vibration bands at 1345 and 1603 cm^−1^ become more intense upon discharge, consistent with NH_4_^+^ intercalation, and decrease during charging, indicating NH_4_^+^ deintercalation. These results highlight the reversibility of ion storage in mixed electrolytes.

**Figure 3:**
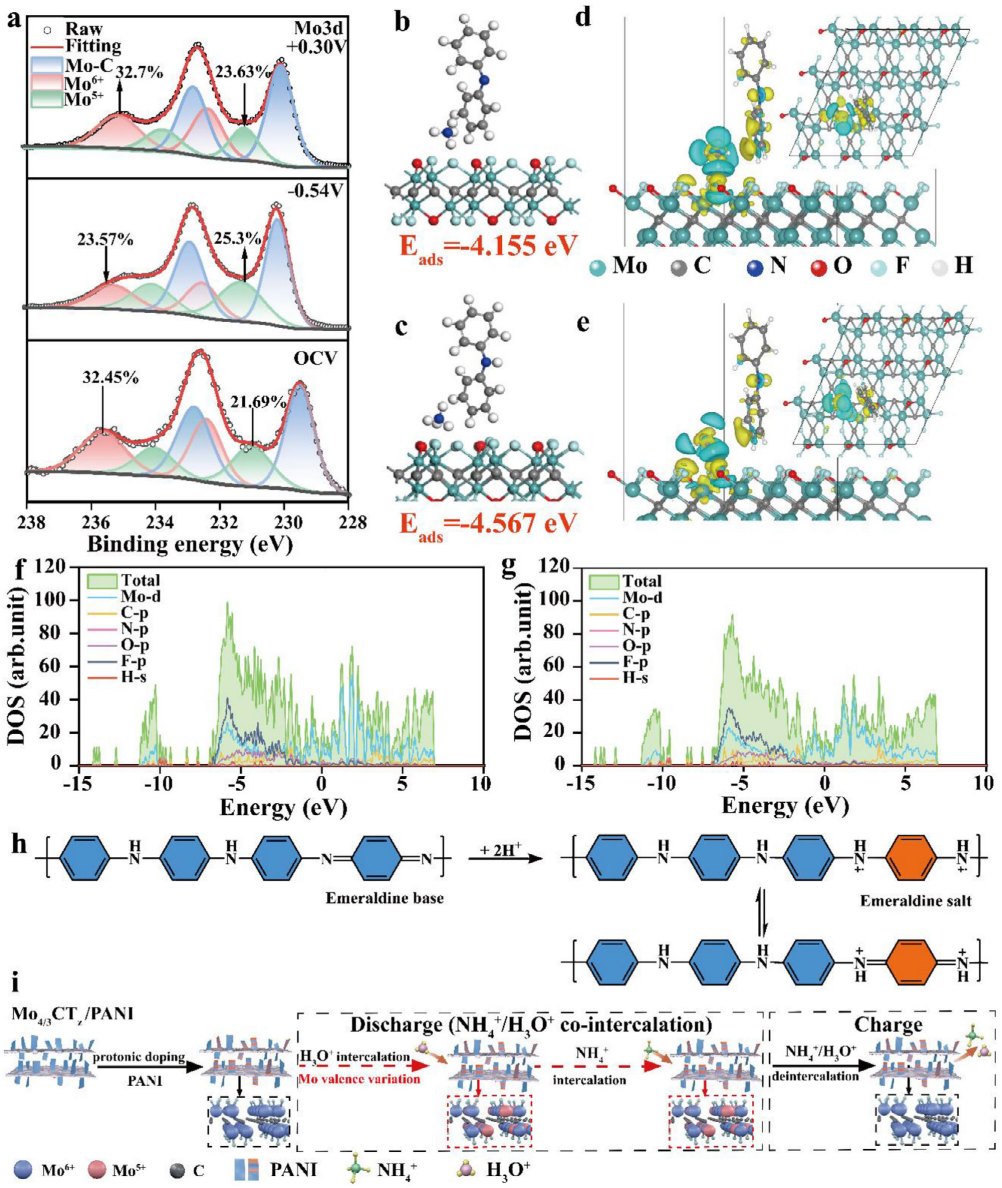
Proton‐assisted NH_4_^+^ storage mechanism. a) The ex situ XPS spectra of Mo 3d in 1–0.1 m mixed electrolyte. Optimized side‐viewed configuration with corresponding adsorption energy in the +6 valent Mo b) and +5 valent Mo c) of the composite. Optimized side‐viewed configuration with corresponding charge density difference in the +6 valent Mo d) and +5 valent Mo e) of the composite. DOS of the composite in the +6 valent Mo f) and +5 valent Mo g) state, respectively. Scheme displaying the proton activation of PANI h) and the proton‐assisted NH_4_^+^/H_3_O^+^ co‐intercalation mechanism in composite materials i).

To clarify the effect of proton additives on NH_4_^+^ storage, a combination of experimental characterizations and theoretical calculations was performed. First, to gain insight into the redox behavior during cycling, ex situ X‐ray photoelectron spectroscopy (XPS) was employed to monitor the valence state evolution during the ion intercalation/de‐intercalation process. As shown in **Figures** **3**a and S10 (Supporting Information), high‐resolution Mo 3d spectra were collected in 1–0.1 m, 0.1 m H_2_SO_4_, and 1 m (NH_4_)_2_SO_4_ electrolytes. The deconvoluted spectra reveal three distinct doublets corresponding to Mo^6+^, Mo^5+^, and Mo─C species. Notably, in both the 1–0.1 m and 0.1 m H_2_SO_4_ electrolytes, the relative intensity of Mo^5+^ increases during discharge and decreases upon charging, indicating a reversible redox transition. In contrast, negligible valence change is observed in the pure 1 m (NH_4_)_2_SO_4_ electrolyte, highlighting the essential role of protons in activating the redox behavior of Mo (Table S3, Supporting Information). Complementary N 1s XPS spectra (Figure S11, Supporting Information) were collected at the pristine, fully discharged, and fully charged states of the M:P = 5:1 composite. In both the 1–0.1 m and 0.1 m H_2_SO_4_ electrolytes, the initial intensity of the ─NH^+^· peak is significantly higher than in the pure 1 m (NH_4_)_2_SO_4_, confirming proton doping of PANI by sulfuric acid, which enhances conductivity. Upon discharge, the ─NH^+^· signal decreases while the ─NH─ peak increases, indicating NH_4_^+^ intercalation and PANI reduction. These features are fully recovered after charging, reflecting excellent redox reversibility, which is also supported by the in situ FTIR results (Figure S12, Supporting Information). Based on the aforementioned results, it can be concluded that the presence of protons in the 1–0.1 m electrolyte not only induces valence changes in Mo sites within MXene but also dope PANI, thereby effectively promoting the ion intercalation, and ultimately enhancing the charge storage performance of the electrode material.

To elucidate the role of proton induction in enhancing ion storage performance, first‐principles calculations were performed based on the structural characteristics of the M:P = 5:1 composite. Given the sheet‐like morphology of PANI on the Mo_4/3_CT_z_ surface, this configuration was adopted in the computational model, and two models were constructed with Mo atoms predominantly in the +6 or +5 valence state, respectively (Figure 3b,c; Figure S13, Supporting Information). The results show that in both models, NH_4_^+^ preferentially adsorbs at the composite interface, with adsorption energies of −4.567 and −4.155 eV for the +5 and +6 valence models, respectively, indicating stronger ion binding affinity in the +5 state. The preferential interfacial adsorption is likely to result from the synergistic effects of local charge distribution, surface polarity, and hydrogen bonding at the interface. Further insight is gained from charge density difference analysis (Figure 3d,e), which reveals significant interfacial charge transfer upon NH_4_^+^ adsorption, where NH_4_^+^ acts as an electron donor and both PANI and MXene serve as electron acceptors. Compared to the +6 model, the +5 configuration exhibits lower adsorption energy and more substantial interfacial electron redistribution, confirming its enhanced ion‐binding capability, which is consistent with its more favorable adsorption energy of −4.567 eV discussed above. Moreover, the total and projected density of states (DOS) profiles (Figure 3f,g; Figures S14 and S15, Supporting Information) confirm metallic conductivity for both models, with Mo‐d orbitals dominating near the Fermi level. Strong interactions among Mo‐d, O‐p, C‐p, and F‐p orbitals are also evident below the Fermi level. Notably, it shows that the Mo valence variation induces significant orbital charge redistribution. The +5 model exhibits a smoother DOS profile with more delocalized states, which facilitates charge transport, reduces local polarization, and improves dynamic charge equilibrium during ion intercalation, thereby collectively enhancing energy storage performance.

Finally, by integrating both experimental and theoretical findings, the overall ion storage mechanism of the composite in the 1–0.1 m electrolyte is schematically illustrated in Figure 3h,i. Initially, protons from sulfuric acid dope PANI, converting it from an insulating emeraldine base into a semiconducting emeraldine salt. This process activates the redox activity of PANI and enhances the electronic conductivity of the composite (as supported by EIS results in Figure S16 (Supporting Information) for 1 m (NH_4_)_2_SO_4_ versus 1–0.1 m electrolytes). During discharge, protons preferentially adsorb at the composite material and concurrently trigger redox transitions at Mo sites. The resulting increase in Mo^5+^ content further enhances NH_4_^+^ adsorption at the interface, enabling the co‐intercalation of NH_4_^+^ and H_3_O^+^ ions. This synergistic mechanism underlies the pronounced pseudocapacitive behavior observed in the mixed electrolyte. Upon charging, the Mo^5+^/Mo^6+^ ratio reverts to its original state, and the intercalated ions are released, demonstrating excellent electrochemical reversibility. Therefore, protons in the mixed electrolyte play a dual role: i) doping PANI to enhance electronic conductivity and pseudocapacitive contribution, and ii) facilitating the reversible redox transitions of Mo in MXene, thereby accelerating interfacial NH_4_^+^ adsorption and intercalation kinetics. These synergistic effects collectively contribute to the superior ammonium storage performance of the M:P = 5:1 composite.

**Figure 4:**
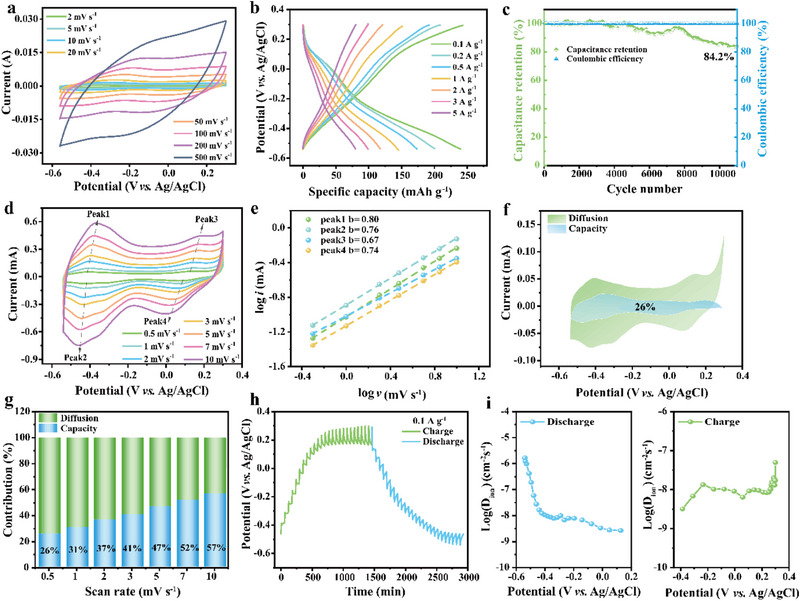
Electrochemical performance and quantitative capacity analysis of M:P = 5:1 composite in the 1‐‐0.1 m mixed electrolyte. a) CV curves at various scan rates ranging from 2 to 500 mV s^−1^. b) GCD curves at different current densities. c) Long‐term cycling performance of the composite at 1 A g^−1^. d) CV curves at various scan rates from 0.5 to 10 mV s^−1^. e) The corresponding log (peak current) versus log (scan rate) plots for M:P = 5:1 electrode. f) Capacitance and diffusion contribution at the scan rate of 0.5 mV s^−1^. g) Capacitive and diffusion contribution of M:P = 5:1 composite at different scan rates. h) Galvanostatic intermittent titration technique (GITT) profiles. i) Ion diffusion coefficient of the M:P = 5:1 composite during charge and discharge calculated from the GITT curve.

The electrochemical performance of the M:P = 5:1 composite in the optimized mixed electrolyte (1–0.1 m) was systematically evaluated. As illustrated in **Figure** **4**a, the CV curves recorded at various scan rates display two well‐defined pairs of redox peaks, confirming the material's excellent pseudocapacitive behavior. Notably, the CV curves retain their shape as the scan rate increases, while the enclosed area expands progressively, indicating fast and efficient charge storage kinetics. Correspondingly, the GCD profiles (Figure 4b) display two distinct discharge plateaus, which align well with the redox peaks observed in the CV curves. Notably, the electrode achieves a high specific capacity of 245 mAh g^−1^ at 0.1 A g^−1^ in 1–0.1 m electrolyte, significantly surpassing its behavior in the pure (NH_4_)_2_SO_4_ electrolyte. The long‐term cycling stability of the M:P = 5:1 composite was evaluated at 1.0 A g^−1^. As shown in Figure 4c, the M:P = 5:1 composite maintains a high‐capacity retention of 84.2% and a coulombic efficiency of ≈100% after 11 000 charge/discharge cycles, underscoring its excellent long‐term stability compared to 1 m (NH_4_)_2_SO_4_ (Figure S17, Supporting Information). This exceptional performance is mainly attributed to the structural robustness of the composite and the proton‐enabled synergistic activation of pseudocapacitive behavior in both the PANI and MXene components.

To gain deeper insight into the electrochemical kinetics underlying the superior ion storage performance of the M:P = 5:1 composite in the 1–0.1 m mixed electrolyte, CV measurements were conducted over a scan rate range of 0.5–10 mV s^−1^ (Figure 4d). The CV profiles exhibit minimal peak shifts and maintain a consistent shape even at higher scan rates, indicating favorable kinetics and a low ion diffusion barrier within the composite. The relationship between current (*i*) and scan rate (*v*) follows the power‐law expression *i* = *av^b^* ^,[^ ^29^ ^]^ where b reflects the charge storage mechanism: b = 0.5 indicates diffusion‐controlled behavior, while b = 1 corresponds to a capacitive‐dominated process. The calculated b‐values for the four redox peaks are 0.80, 0.79, 0.67, and 0.74 (Figure 4e), suggesting a combined contribution from both diffusion and capacitive processes. This hybrid charge storage mechanism accounts for the composite's notable rate capability and pseudocapacitive behavior. The enhanced performance is primarily attributed to the engineered architecture, where abundant metal vacancies in Mo_4/3_CT_z_ serve as active sites, and the wrinkled arrangement of PANI nanosheets on the MXene surface enables efficient ion transport. To further quantify the relative contributions of capacitive and diffusion‐controlled processes, the current response at a given potential (𝑉) can be deconvoluted using the expression *i*(*V*) = *k*_1_ + *k*_2_ *v* ^1/2^, where 𝑘_1_ and 𝑘_2_ are the coefficients for capacitive and diffusion‐controlled processes, respectively.^[^ ^30^ ^]^ At a scan rate of 0.5 mV s^−1^, the capacitive contribution constitutes ≈26% of the total capacity (Figure 4f). A broader analysis across various scan rates (Figure 4g) reveals that while diffusion dominates at lower rates, the capacitive contribution increases progressively with scan rate, demonstrating enhanced kinetics and faster charge storage at higher scan rates. Additionally, the galvanostatic intermittent titration technique (GITT) was employed to evaluate the diffusion coefficient of NH_4_^+^/H_3_O^+^ ions (Figure 4h). The resultant diffusion coefficient falls within the range of 10^−9^–10^−7^ cm^2^ s^−1^ (Figure 4i), further confirming the excellent ion transport kinetics of the M:P = 5:1 composite.

**Figure 5:**
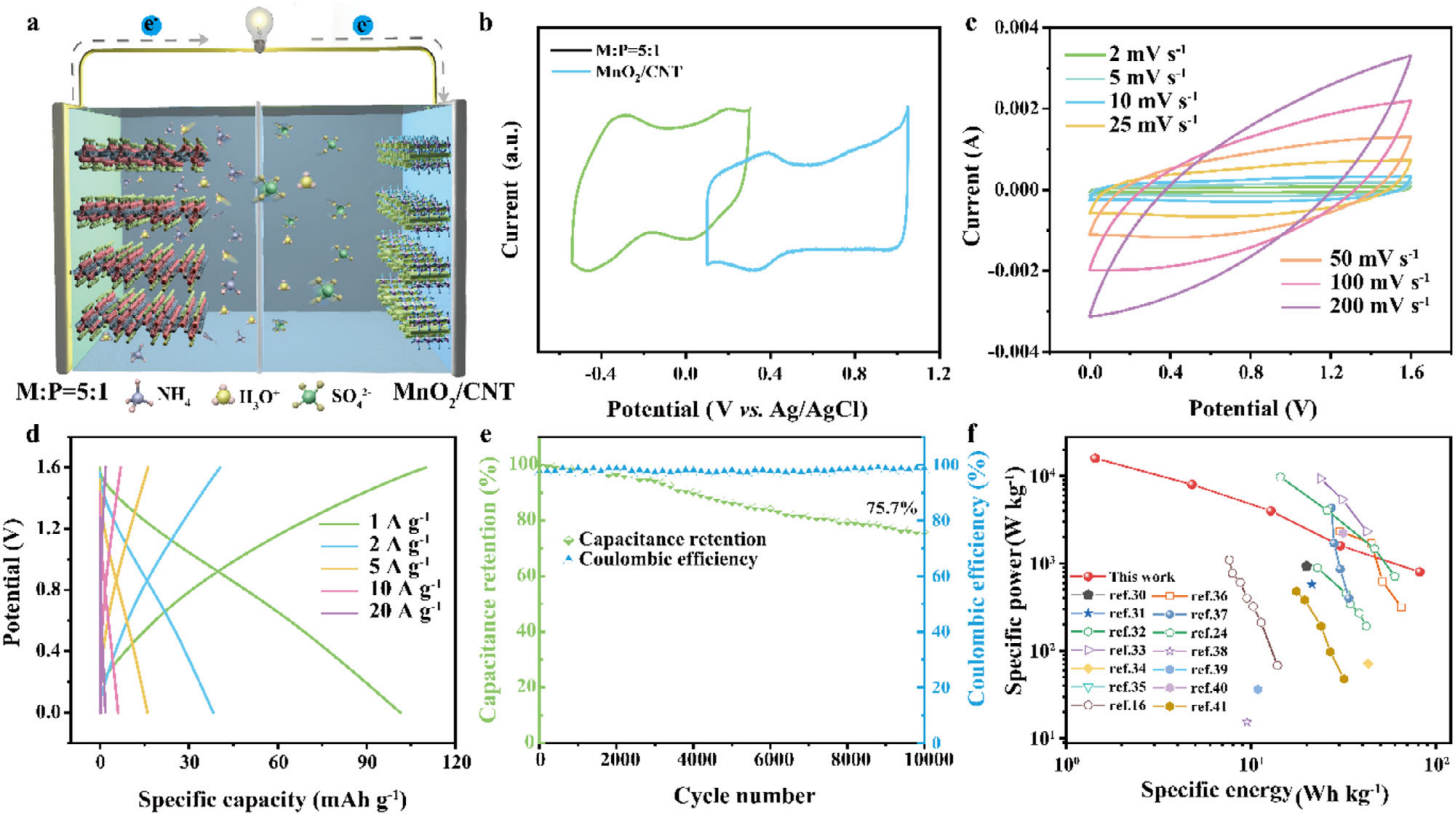
The electrochemical properties of the full cell. a) Schematic illustration of the MnO_2_/CNTs||Mo_4/3_CT_z_//PANI cell. b) CV curves of the individual MnO_2_/CNTs and Mo_4/3_CT_z_/PANI electrodes at 10 mV s^−1^. c) CV curves of the full cell recorded at different scan rates. d) GCD curve under different current densities. e) Long‐term stability at 1 A g^−1^. f) Ragone plot comparing energy and power densities with previously reported AAIB systems.

The full AAIB was assembled using the M:P = 5:1 composite as the anode, an MnO_2_/CNTs as the cathode, and a 1–0.1 m mixed electrolyte (**Figure** **5**a). The synthesis and characterization of MnO_2_ and MnO_2_/CNTs are provided in Figure S18 (Supporting Information). Charge balance between the electrodes was achieved by adjusting their respective mass loadings. As shown in Figure 5b, the CV curves of both electrodes at a scan rate of 10 mV s^−1^ exhibit well‐aligned electrochemical behavior, indicating good compatibility and synergistic operation. The assembled full cell operates over an expanded voltage window of 1.6 V, as evidenced by the CV curves recorded at various scan rates (Figure 5c; Figure S19a, Supporting Information). In addition, the highly reversible ion intercalation/deintercalation in the mixed electrolyte facilitates a nearly symmetrical GCD profile (Figure 5d), delivering a high specific capacity of 101.4 mAh g^−1^ at a current density of 1 A g^−1^. The Nyquist plot and equivalent circuit of the AAIB (Figure S19b, Supporting Information and inset) reveal a low equivalent series resistance (R_s_) of 8.46 Ω and a charge transfer resistance (R_ct_) of 1.25 Ω, indicating efficient ion transport and low internal resistance within the device. The AAIB also exhibits excellent cycling stability. As shown in Figure 5e, the full cell retains 75.7% of its initial capacity after 10 000 cycles at 1 A g^−1^, while maintaining a Coulombic efficiency close to 100%, highlighting the long‐term durability of the M:P = 5:1 composite. Furthermore, the energy and power output of the full cell are illustrated in the Ragone plot (Figure 5f), along with comparisons to previously reported MXene‐based AAIBs.^[^ ^16^, ^27^, ^31^, ^32^, ^33^, ^34^, ^35^, ^36^, ^37^, ^38^, ^39^, ^40^, ^41^, ^42^ ^]^ The MnO_2_/CNTs||M:P = 5:1 device delivers a maximum energy density of 81.6 Wh kg^−1^ and a wide power density range from 800 to 16 000 W kg^−1^, outperforming many state‐of‐the‐art aqueous ammonium‐ion systems (Table S4, Supporting Information).

## 3 Conclusion

In conclusion, an effective strategy for achieving high‐performance non‐metallic NH_4_^+^/H_3_O^+^ co‐intercalation behavior is untangled in Mo_4/3_CT_z_ MXene/PANI composites triggered by simultaneously activating the pseudocapacitive behaviors of both Mo_4/3_CT_z_ MXene and PANI through the introduction of sulfuric acid into the ammonium sulfate electrolyte. This enhanced performance is closely related to the tailored architecture of the composite, where nanosheet‐like PANI structures are grown in situ on the MXene surface, forming a wrinkled morphology that expands the interlayer spacing, facilitates continuous electron/ion transport, and provides favorable sites for NH_4_^+^ adsorption. Beyond the structural advantages, comprehensive experimental and theoretical studies further reveal that protons play two critical roles: i) proton doping of PANI enhances the conductivity and charge carrier mobility of the composite; ii) Proton‐induced reversible Mo valence transitions dynamically modulate the NH_4_⁺ adsorption energy (from −4.155 to −4.567 eV), thereby facilitating both intercalation and deintercalation of NH_4_^+^. Benefiting from this synergistic design, the M:P = 5:1 composite delivers a high specific capacitance of 245 mAh g^−1^ at a current density of 0.1 A g^−1^ in the 1–0.1 m mixed electrolyte. Notably, it maintains a stable capacity retention of 84.2% after 11 000 cycles at 1.0 A g^−1^, significantly outperforming previously reported ammonium ion storage materials. As a proof of concept, the MnO_2_/CNTs||M:P = 5:1 full cell achieves a high energy density of 81.6 Wh kg^−1^ at a power density of 800 W kg^−1^, while retaining 75.5% of its initial capacitance after 10 000 cycles at 1 A g^−1^. These findings provide valuable insights into the development of high‐performance, sustainable non‐metallic ion storage materials and offer a promising path forward for the development of advanced energy storage technologies.

## Conflict of Interest

The authors declare no conflict of interest.

## Supporting information



Supporting Information

## Data Availability

The data that support the findings of this study are available from the corresponding author upon reasonable request.
