# Supplementary material for: Activation of the Pseudocapacitive Behavior of MXene/PANI for High‐Performance Ammonium‐Ion Batteries
Source: Adv Sci (Weinh). 2025 Aug 21;12(43):e11815. doi: 10.1002/advs.202511815 (PMC12631864; doi:10.1002/advs.202511815)
Supplement: Supplementary file 1 — Supporting Information [file ADVS-12-e11815-s001.docx]

Supporting Information

Activation the Pseudocapacitive Behavior of MXene/PANI for High-Performance Ammonium-Ion Batteries

*Yeying Li, Leixi Du, Liping Zhang, Changjie Huang, Justinas Palisaitis, Jingkun Xu, Johanna Rosen*, *Jianxia Jiang^*^, Leiqiang Qin^*^*

Y. Li, L. Du, L. Zhang, J. Xu, J. Jiang

Flexible Electronics Innovation Institute (FEII), Jiangxi Provincial Key Laboratory of Flexible Electronics, Jiangxi Science and Technology Normal University, Nanchang 330013, China

1. mail: [j.jianxia@qq.com](mailto:j.jianxia@qq.com)
2. Li, L. Du

School of Chemistry and Chemical Engineering, Jiangxi Science & Technology Normal University, Nanchang 330013, Jiangxi, China

C. Huang, J. Palisaitis, J. Rosen, L. Qin

Department of Physics, Chemistry and Biology (IFM), Linköping University, Linköping 58183, Sweden

E-mail: [leiqiang.qin@liu.se](mailto:leiqiang.qin@liu.se)

**1. Experimental Section**

**1.1. Materials Synthesis**

*1.1.1 Preparation of few-layer Mo_4/3_CT_z_ suspension*

Few-layer Mo_4/3_CT_z_ suspension was synthesized by etching Mo_4/3_Sc_2/3_AlC MAX powder in 48% HF. Specifically, 1 g of Mo_4/3_Sc_2/3_AlC powder was mixed with 20 mL of 48% HF and stirred at 31°C for 24 hours. The resulting acidic dispersion was then washed with deionized water by centrifugation (6500 rpm for 3 min) repeatedly until the supernatant reached a neutral pH (~7). The wet sediment obtained from this process was dispersed in 5 mL of 40% tetrabutylammonium hydroxide (TBAOH) solution. After an additional round of centrifugation and washing with deionized water, the mixture was centrifuged for 30 minutes at 2500 rpm to remove precipitate. The supernatant collected after this final step yielded the few-layer Mo_4/3_CT_z_ suspension.

*1.1.2 Preparation of polyaniline*

Polyaniline (PANI) was synthesized through chemical oxidation. Specifically, 1 g of aniline monomer was ultrasonically dispersed in 50 mL of 3 M HCl for 10 minutes. Under continuous stirring, a 1 M HCl solution containing 7.3 g of ammonium persulfate (APS) was rapidly added to the dispersion. The mixture was stirred thoroughly for 10 minutes, followed by reaction in an ice bath for 12 hours. The resulting precipitate was washed repeatedly with deionized water and anhydrous ethanol until the supernatant reached a neutral pH (pH = 7). The final product was dried under vacuum at 60°C for 12 hours, yielding dark green polyaniline (PANI).

*1.1.3 Preparation of Mo_4/3_CT_z_/PANI composites with Different Mass Ratios*

Polyaniline (PANI) was synthesized and polymerized in situ on Mo_4/3_CT_z_ flakes via chemical oxidation to prepare Mo_4/3_CT_z_/PANI composites with varying mass ratios. Specifically, aniline monomers (9.8 μL, 19.6 μL, 48.9 μL, and 97.8 μL, corresponding to mass ratios of Mo_4/3_CT_z_ to PANI (M:P) of 10:1, 5:1, 2:1, and 1:1, respectively) were ultrasonically dispersed in deionized water. To each dispersion, 100 mg of Mo_4/3_CT_z_ MXene suspension was added, and the mixture was stirred thoroughly before refrigeration for 15 minutes. Subsequently, a pre-cooled 1 M hydrochloric acid solution containing ammonium persulfate (APS) was added dropwise while maintaining a molar ratio of aniline, hydrochloric acid, and APS at 1:3:0.5. The polymerization reaction was carried out at 0°C for 13 hours. The resulting precipitate was collected and washed with deionized water and anhydrous ethanol until the pH of the supernatant reached 7. Finally, the product was dried under vacuum at 60°C for 12 hours to yield Mo_4/3_CT_z_/PANI composite materials.

*1.1.4. Preparation of MnO_2_-CNTs suspension*

1 g Mn_3_O_4_ powder was added to 10 mL of tetramethylammonium hydroxide (TMAOH, 25 wt.% in H_2_O) and stirred at 80°C for 3 days. The resulting reaction mixture was then washed with ethanol five times by centrifugation at 6000 rpm for 2 minutes each cycle. The precipitate obtained was subsequently washed three times with water. Next, 30 mL of deionized water was added to the washed precipitate, which was then shaken well and sonicated for 20 minutes. The MnO_2_ suspension was obtained by centrifugation at 6000 rpm for 20 minutes. Finally, the obtained MnO_2_ suspension was ultrasonically mixed with carbon nanotubes (CNTs) at a mass ratio of 1:1 to form the MnO_2_-CNTs suspension.

*1.1.5 Preparation of Mo_4/3_CT_z_/PANI composite films*

The Mo_4/3_CT_z_/PANI composite (70 mg), conductive carbon black (20 mg), and polyvinylidene fluoride (PVDF) binder (10 mg) were thoroughly mixed in 350 μL of N-methyl-2-pyrrolidone (NMP) solvent. The resulting slurry was coated onto a clean, dry titanium foil substrate (15 cm × 7 cm × 0.001 cm) and dried under vacuum at 100°C for 12 hours. The Mo_4/3_CT_z_/PANI electrode film was then obtained by cutting the dried coating into 6 mm diameter circles using appropriate cutting tools.

*1.1.6 Preparation of MnO_2_-CNTs film*

The prepared MnO_2_-CNTs suspension in step 1.1.4 was subjected to vacuum filtration. After drying, the MnO_2_-CNTs membrane electrode was obtained by peeling it off from the polypropylene membrane (Celgard 3501). The MnO_2_-CNTs electrode film was then obtained by punching the dried coating into 6 mm diameter films using a hole punch.

*1.1.7 Assembly of MnO_2_/CNTs||Mo_4/3_CT_z_/PANI AAIBs*

All electrodes used were those mentioned in the previous preparation steps, the cathode was the Mo_4/3_CT_z_/PANI film, and the anode was the MnO_2_-CNTs film. The electrolyte solution was tested in a 1 M-0.1 M mixed electrolyte.

**1.2. Materials Characterizations**

Microstructure and morphology were characterized using a Bruker D2 Phaser X-ray diffractometer with Cu-Kα radiation (λ = 1.5406 Å), a Hitachi HT-7820 transmission electron microscope (TEM), and a Zeiss Sigma 300 field-emission scanning electron microscope (SEM). Raman spectra were obtained with a Renishaw in Via spectrometer using a 532 nm laser. The chemical composition of samples was assessed by a Thermo Scientific K-Alpha X-ray photoelectron spectrometer (XPS) and energy-dispersive X-ray spectroscopy (EDS). Fourier transform infrared (FTIR) spectra were recorded using a Nicolet Nexus 670 spectrometer. Electrochemical quartz crystal microbalance (EQCM) experiments were performed using a commercial Q-Sense Explore system (Biolin Scientific AB, Sweden). The composite electrode slurry was sprayed onto titanium and gold-coated AT-cut quartz crystal sensor chips and then dried at room temperature. EQCM measurements were conducted during cyclic voltammetry (CV) tests using an electrochemical workstation (CHI660E, CH Instruments, Shanghai, China). Atomic-resolution scanning transmission electron microscopy (STEM) imaging was performed in the Linköping double-corrected FEI Titan^3^ 60-300, operated at 300 kV.

**1.3 Electrochemical measurements**

The electrochemical properties of prepared samples were tested in an electrochemical workstation (VSP, Bio-Logic, France) using a three-electrode system consisting of cyclic voltammetry (CV), constant current charge/discharge (GCD), electrochemical impedance spectroscopy (EIS), and constant current cycling. The three-electrode system consists of working electrode (active material: acetylene black: PVDF = 7:2:1), reference electrode (Ag/AgCl electrode), counter electrode (activated carbon) and an electrolyte. Different electrolytes are used for testing:1 M (NH_4_)_2_SO_4_, 0.1 M H_2_SO_4_, 1 M (NH_4_)_2_SO_4_ + 0.01 M H_2_SO_4_, 1 M (NH_4_)_2_SO_4_ + 0.1 M H_2_SO_4_, M (NH_4_)_2_SO_4_ + 1 M H_2_SO_4_, and labeled as 1 M (NH_4_)_2_SO_4_, 0.1 M H_2_SO_4_, 1 M-0.01 M, 1 M-0.1 M, 1 M-1 M, respectively. The EIS are obtained in the frequency range from 0.01 Hz to 1000 kHz. The constant current intermittent titration technique (GITT) is performed with a current pulse of 3.8 mA g^-1^ for 1 minute, followed by a relaxation period of 30 minutes.

In a three-electrode testing system, the specific capacitance was calculated from CV curves according to equation (1), and from GCD curves using Equation (2).

$C_{s}=\frac{\int Idt}{ms}$ (1)

Where Cs (mAh g^-1^) is the mass specific capacitance;

I (A g^-1^) is the current density;

m(g) is the mass of the active material;

s (V s^-1^) is the scanning rate;

△t (s) is the discharge time.

$C_{s}=\frac{I\Delta t}{m}$ (2)

Where I (mA) and △t (h) represented the discharge current and discharge time, respectively; m (g) is the mass of the active material.

For the MnO_2_/CNTs||Mo_4/3_CT_z_/PANI AIBs, the mass ratios of the positive and negative electrodes were balanced according to the following Equation (3):

$\frac{m_{+}}{m_{-}}=\frac{C_{-}{\Delta V}_{-}}{C_{+}{\Delta V}_{+}}$ (3)

The energy density (Wh kg^-1^) and power density (W kg^-1^) were calculated as follows:

$E=\frac{Cs\Delta V^{2}}{2*3.6}$ (4)

$P=\frac{E*3600}{t}$ (5)

where E is the energy density in Wh g^-1^,

C_v_ is the capacitance obtained from galvanostatic charge/discharge curves using Equation (2), expressed in F g^-1^;

ΔE is the operating voltage window in volts;

P is the power density in W kg^-1^;

t is the discharge time in hours.

**1.4** **Simulation details and methodology**

We employ VASP 6.1.0 code^[1,2]^ to implement density functional theory (DFT) calculations. The exchange and correlation interaction are described by Perdew-Burke-Ernzerhof (PBE) method^[3,4]^ of generalized gradient approximation (GGA) functional. We choose the model of Mo_4/3_CT_z_ (5.154 Å×5.191 Å, γ=62.29°) with surface O and F passivation. To control the adsorption density and prevent it from becoming excessively large, a 3×3×1 supercell was employed. One unit of polyaniline was selected to put on the surface of Mo_4/3_CT_z_. Vacuum layers with a thickness of 15 Å are set along vertical direction to avoid periodic effects. The Van der Waals interaction is handled by empirical DFT-D3 (BJ) correction.^[5,6]^ Spin-polarization effect is also taken into consideration due to the existence of Mo element. The cutoff energy is set to be 400 eV. An adequate Gamma-center 2×2×1 k-mesh is utilized for optimization. The criteria of energy convergence and force convergence are set to 10^-4^ eV and 0.02 eV/Å. Valence electron were induced: H (1s^1^), C (2s^2^ and 2p^2^), N (2s^2^ and 2p^3^), O (2s^2^ and 2p^4^), F (2s^2^ and 2p^5^) and Mo (5s^1^ and 4d^5^). All the numerical accuracy has been carefully tested.

**2. Result Section**

**
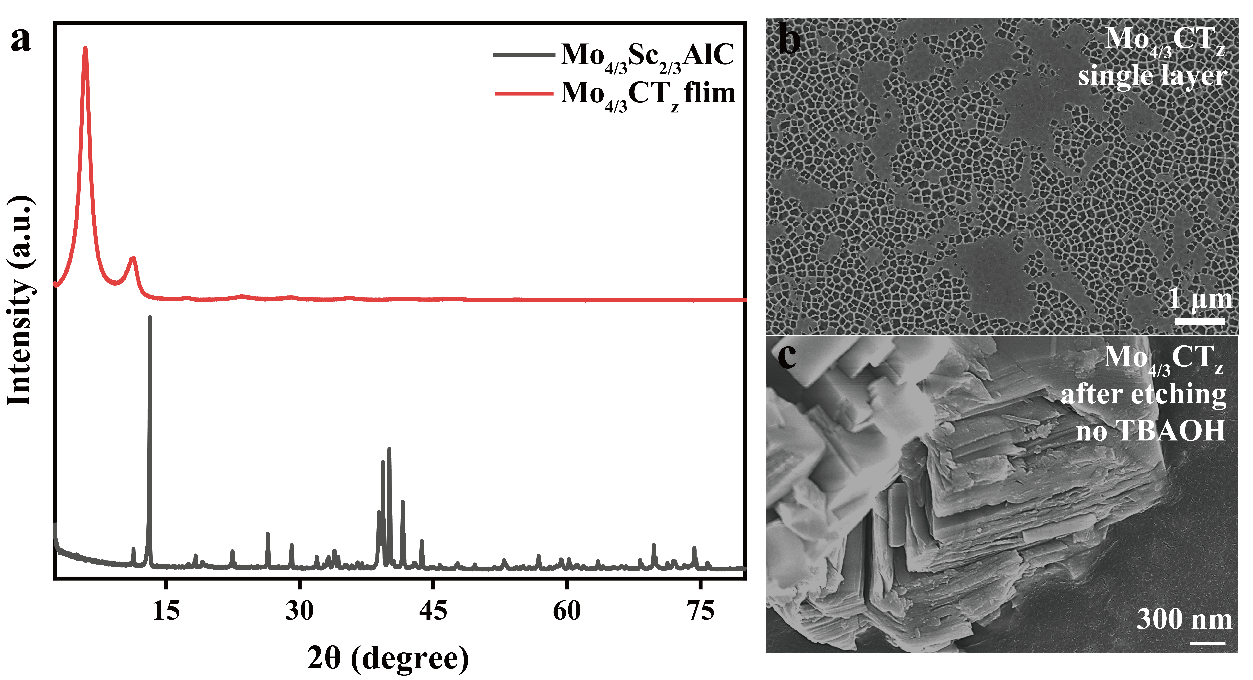
**

**Figure S1.** (a)XRD pattern of Mo_4/3_Sc_2/3_AlC and Mo_4/3_CT_z_ film. SEM images of (b) Mo_4/3_CT_z_ single layer and (c) Multi-layer Mo_4/3_CT_z_ before TBAOH intercation.


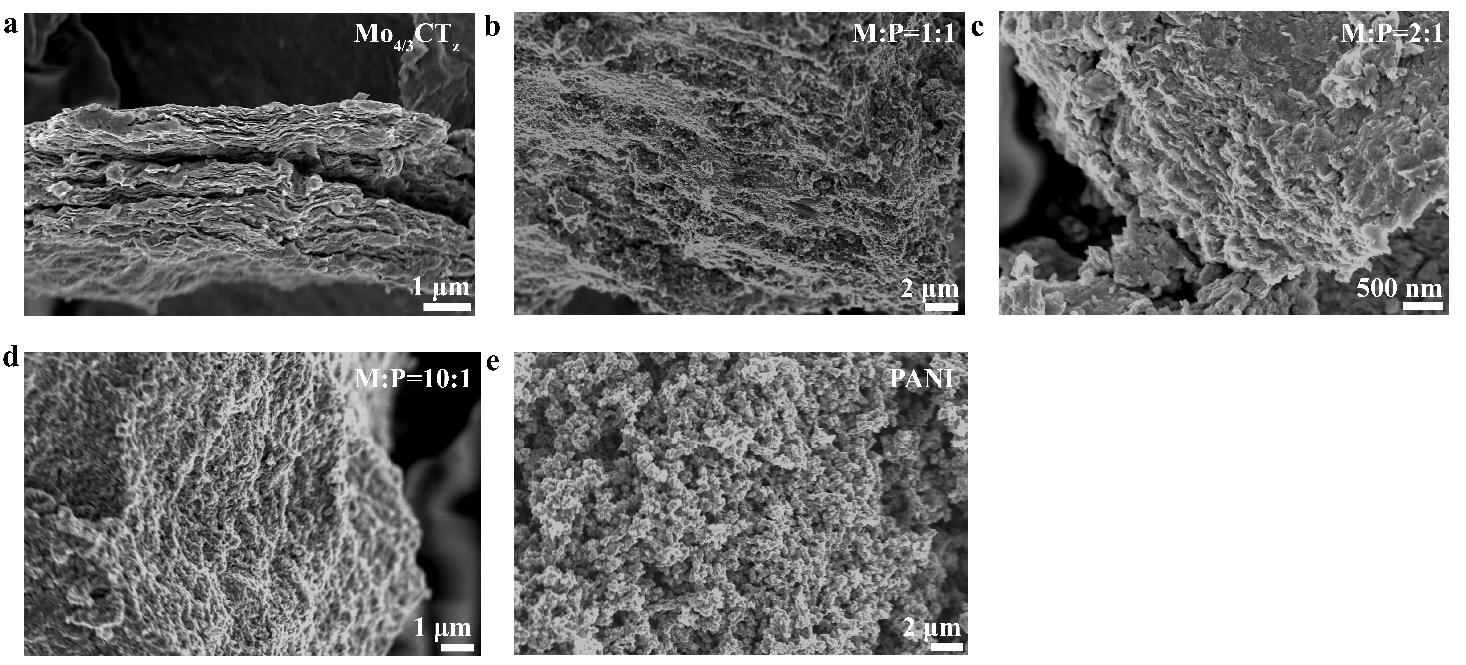


**Figure S2.** SEM images of (a) Mo_4/3_CT_z_, (b) M:P=1:1, (c) M:P=2:1, (d) M:P=10:1, (e) PANI.

**
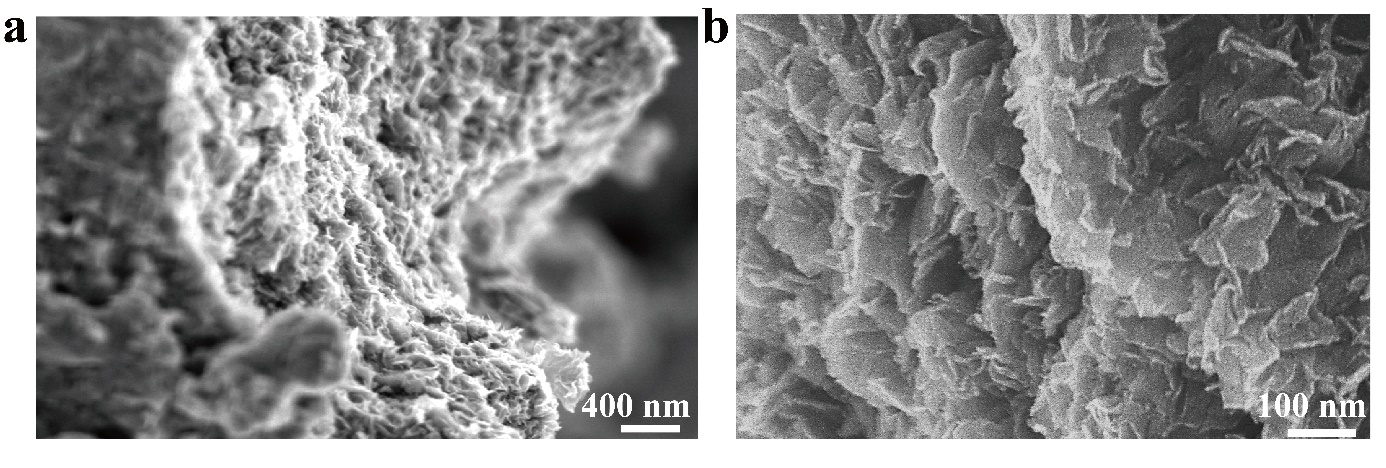
**

**Figure S3.** (a) and (b) SEM images of the M:P = 5:1 composite.


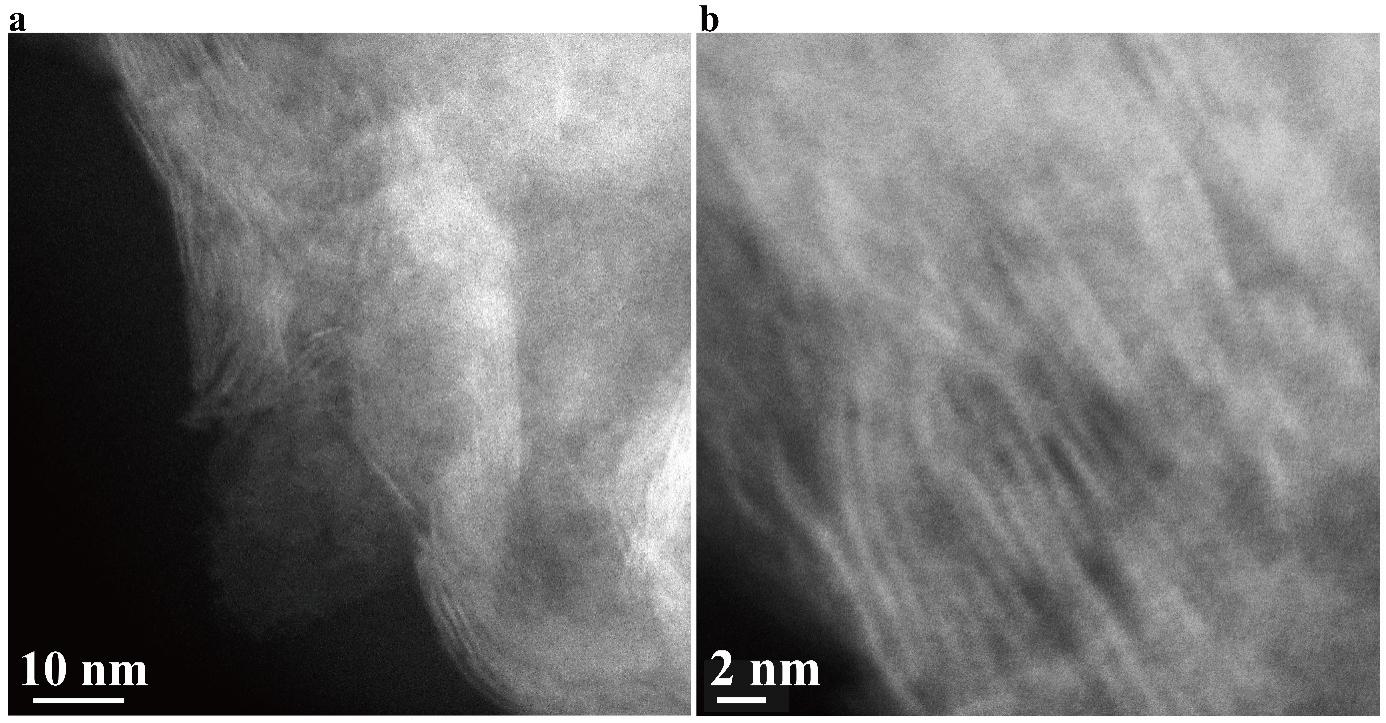


**Figure S4.** (a) and (b) STEM image of the M:P = 5:1 composite.


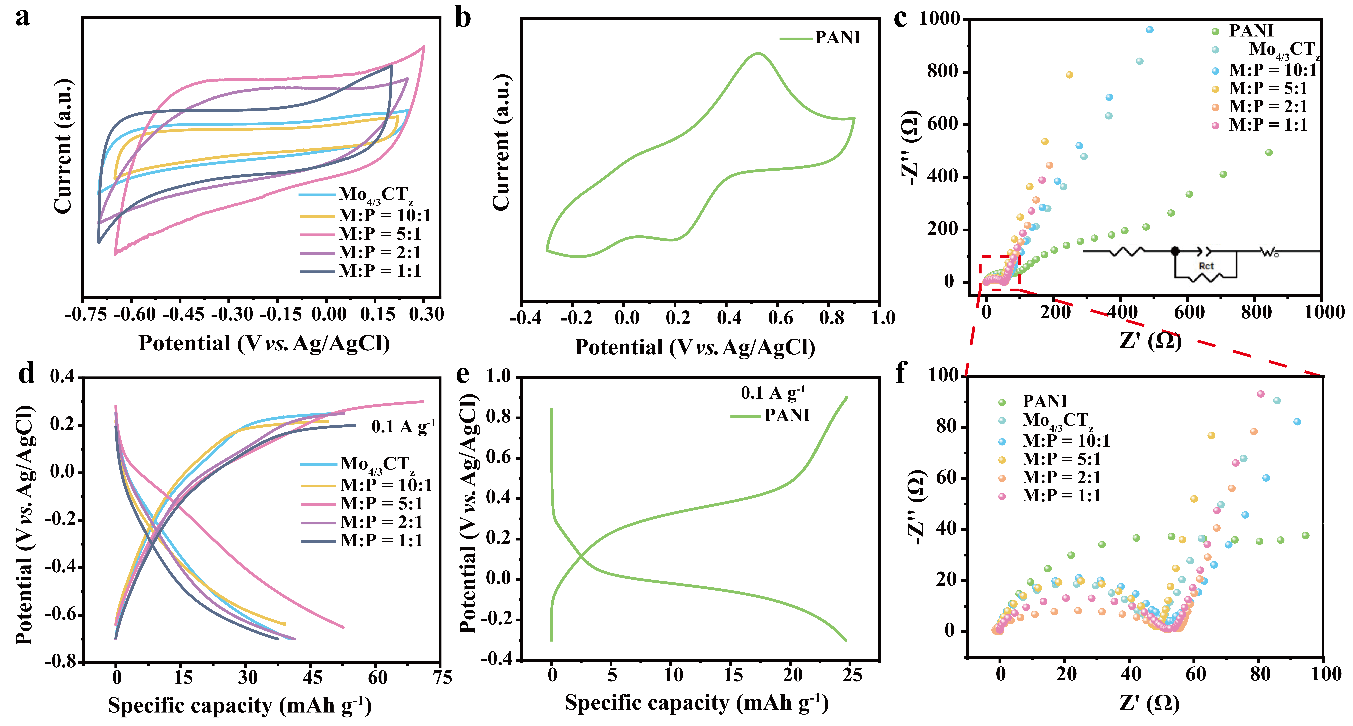


**Figure S5.** Electrochemical performance test of Mo_4/3_CT_z_, PANI and Mo_4/3_CT_z_/PANI composite films in 1 M (NH_4_)_2_SO_4_. (a) and (b) The CV curves at a scan rate of 10 mV s^-1^. (c) The Electrochemical impedance. (d) and (e) The GCD curve at current density of 0.1 A g^-1^. (f）Local magnification of the electrochemical impedance.


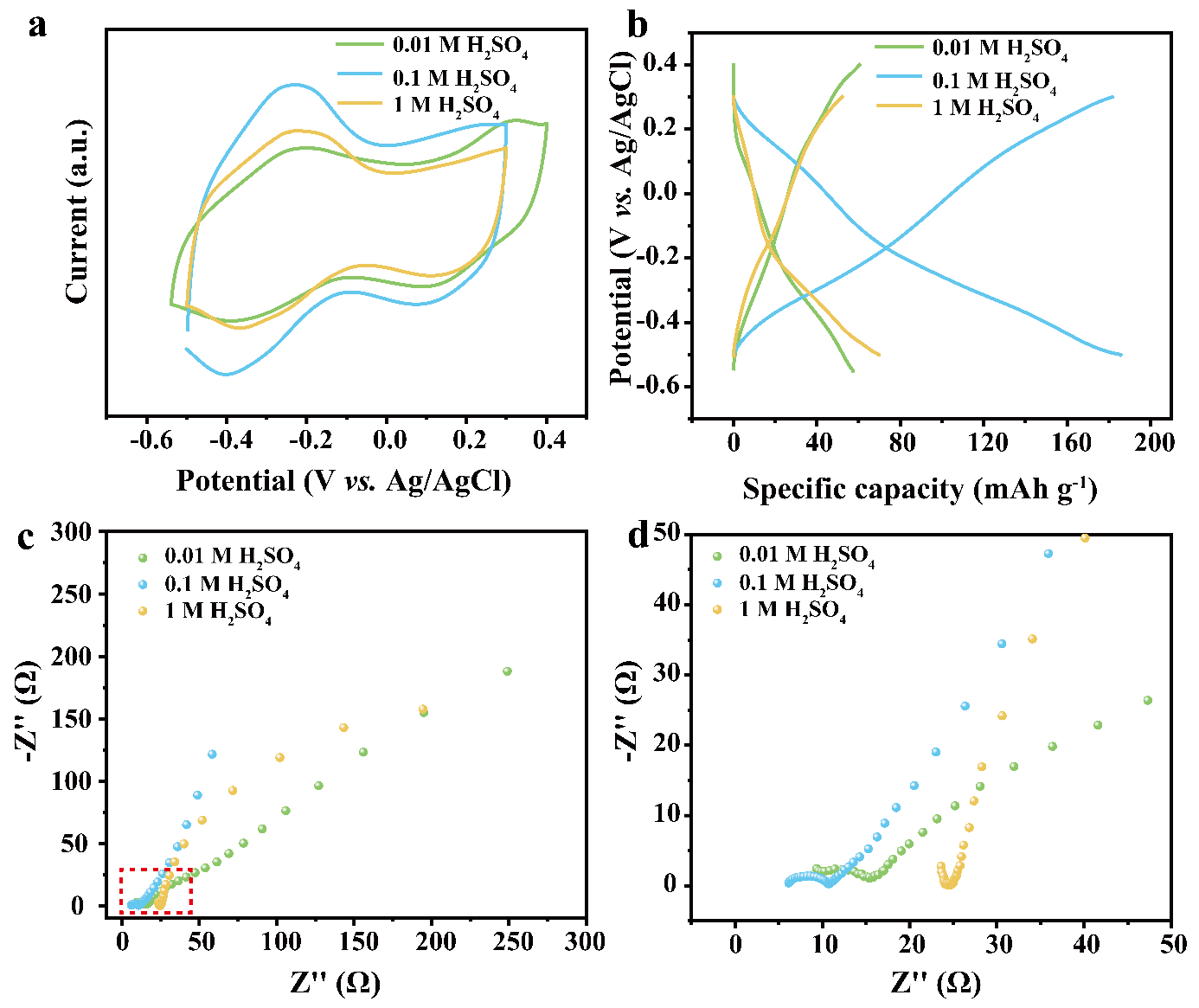


**Figure S6.** The Electrochemical performance of M:P = 5:1 composite under different sulfuric acid concentrations. (a) Cyclic voltammetry curves at a scan rate of 10 mV s^-1^. (b) Galvanostatic charge-discharge curve at 0.1 A g^-1^. (c) EIS characterization and (d) local magnification.


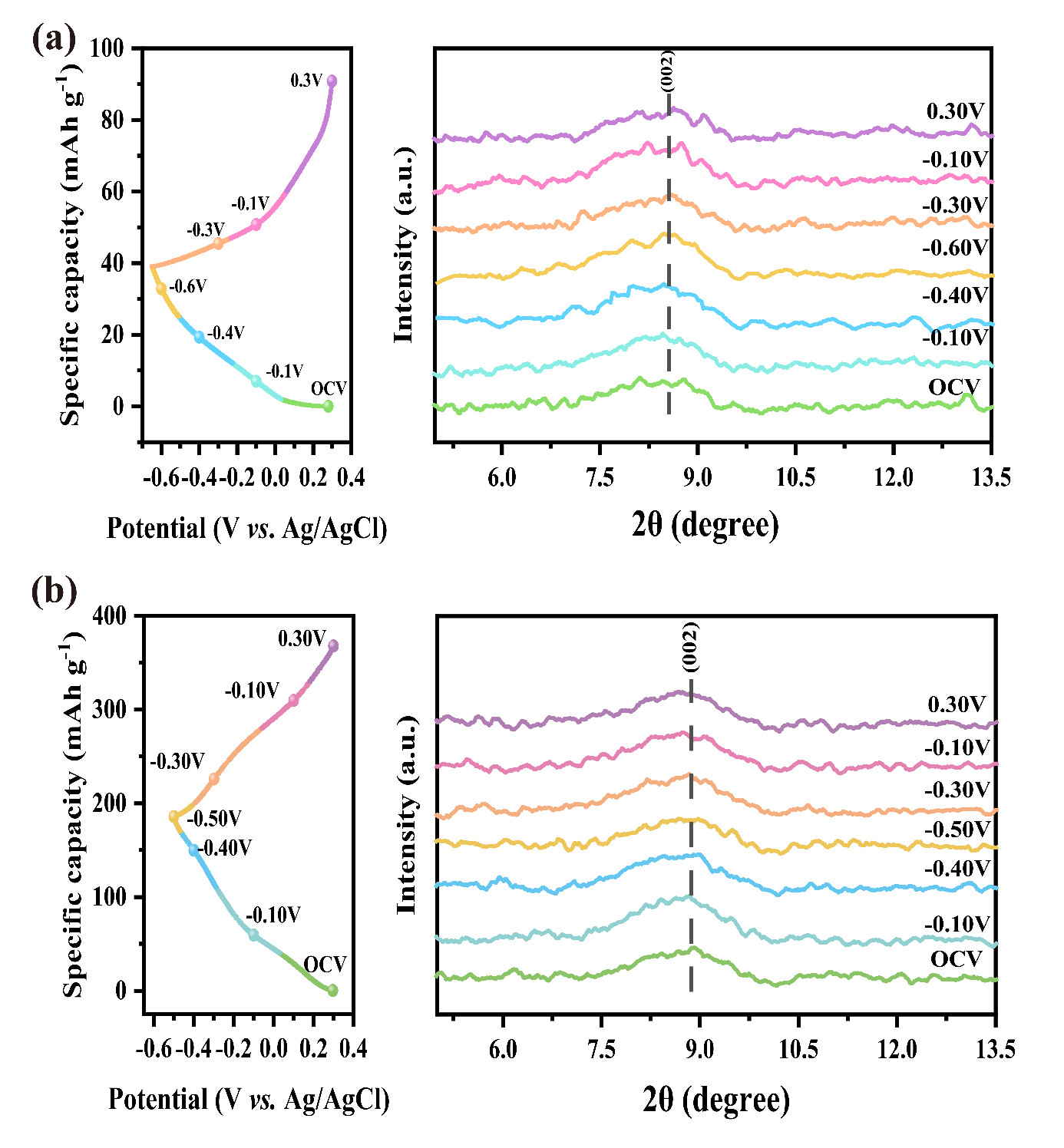
**Figure S7.** Ex situ XRD patterns of the M:P = 5:1 composite at different charge and discharge states in (a) 1 M (NH_4_)_2_SO_4_ and (b) 0.1 M H_2_SO_4_ electrolytes.


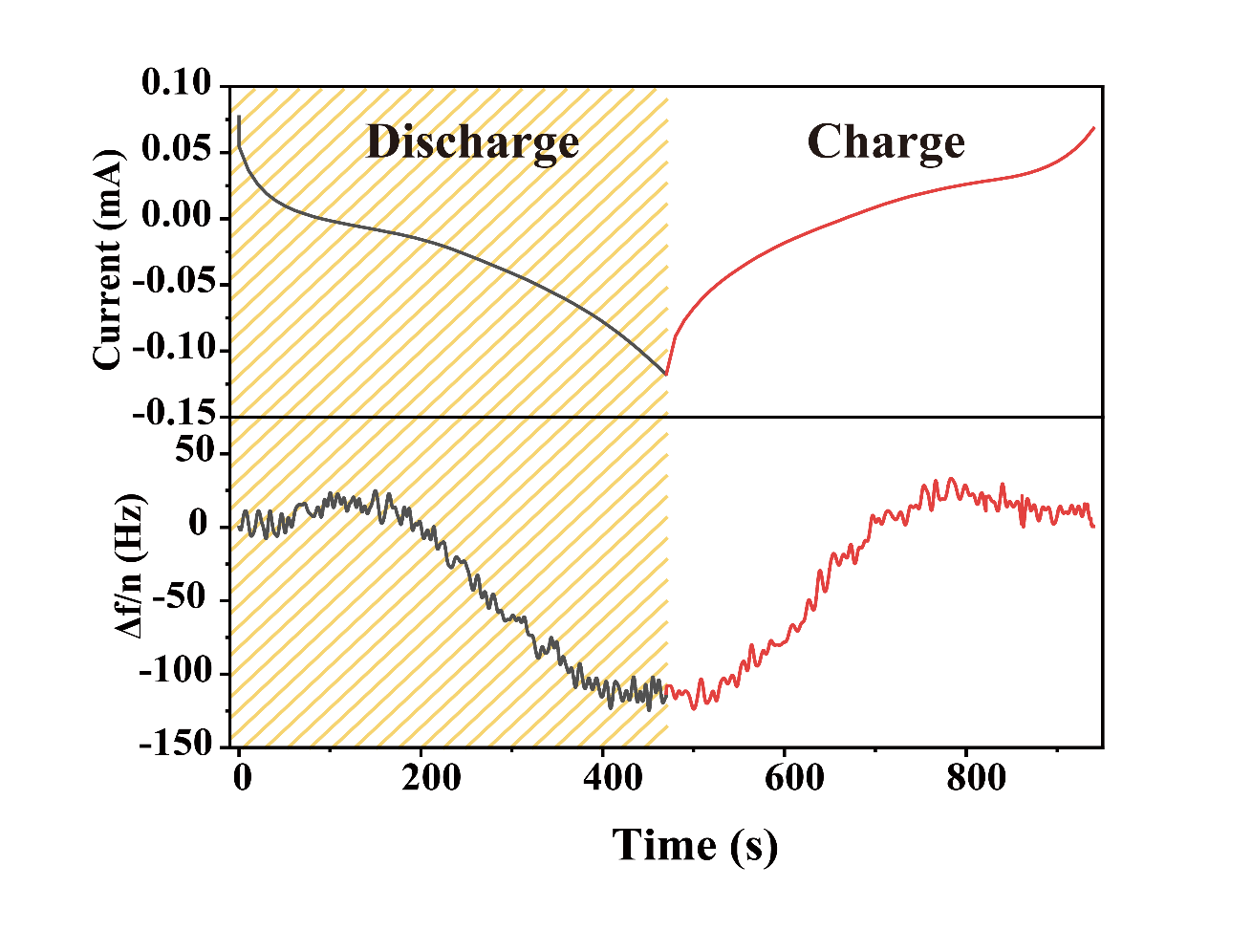


**Figure S8.** Time-resolved CV current (top) and corresponding frequency shift (Δf/n, bottom) for the M:P = 5:1 thin-film electrode recorded at a scan rate of 10 mV s^-1^ in 1 M (NH_4_)_2_SO_4_ electrolyte.


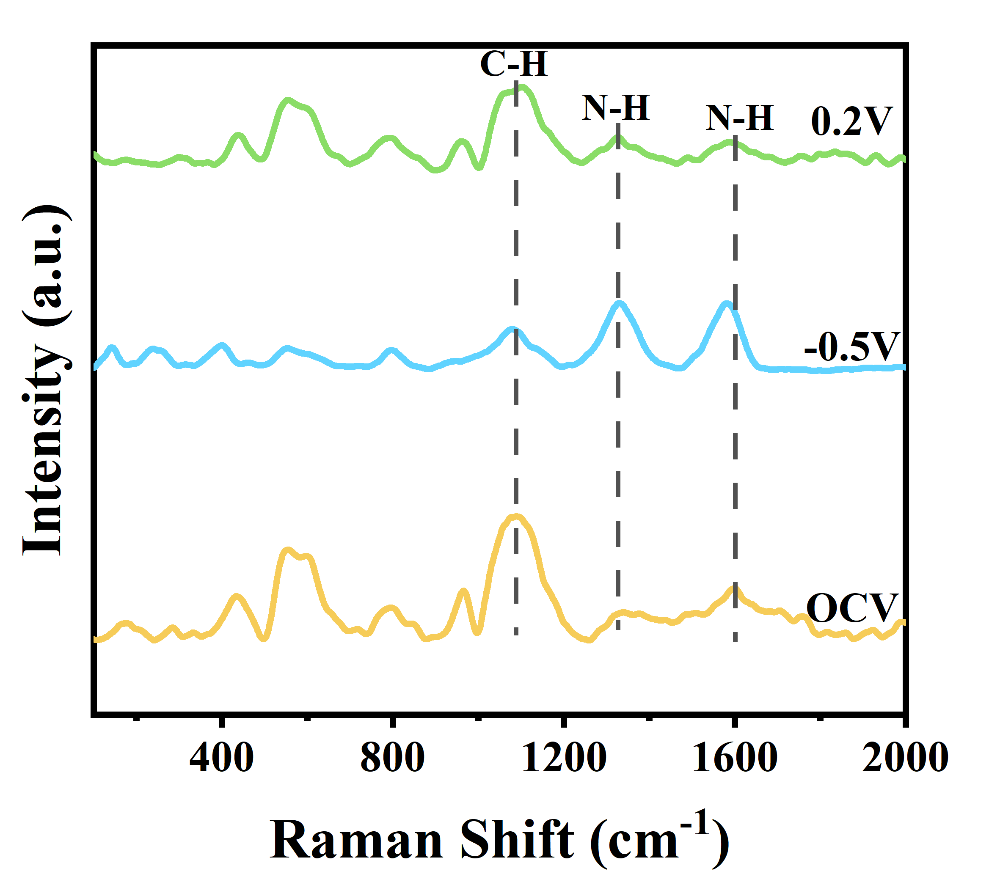


**Figure S9.** In situ Raman spectra of the M:P = 5:1 composite collected at different charge and discharge states.


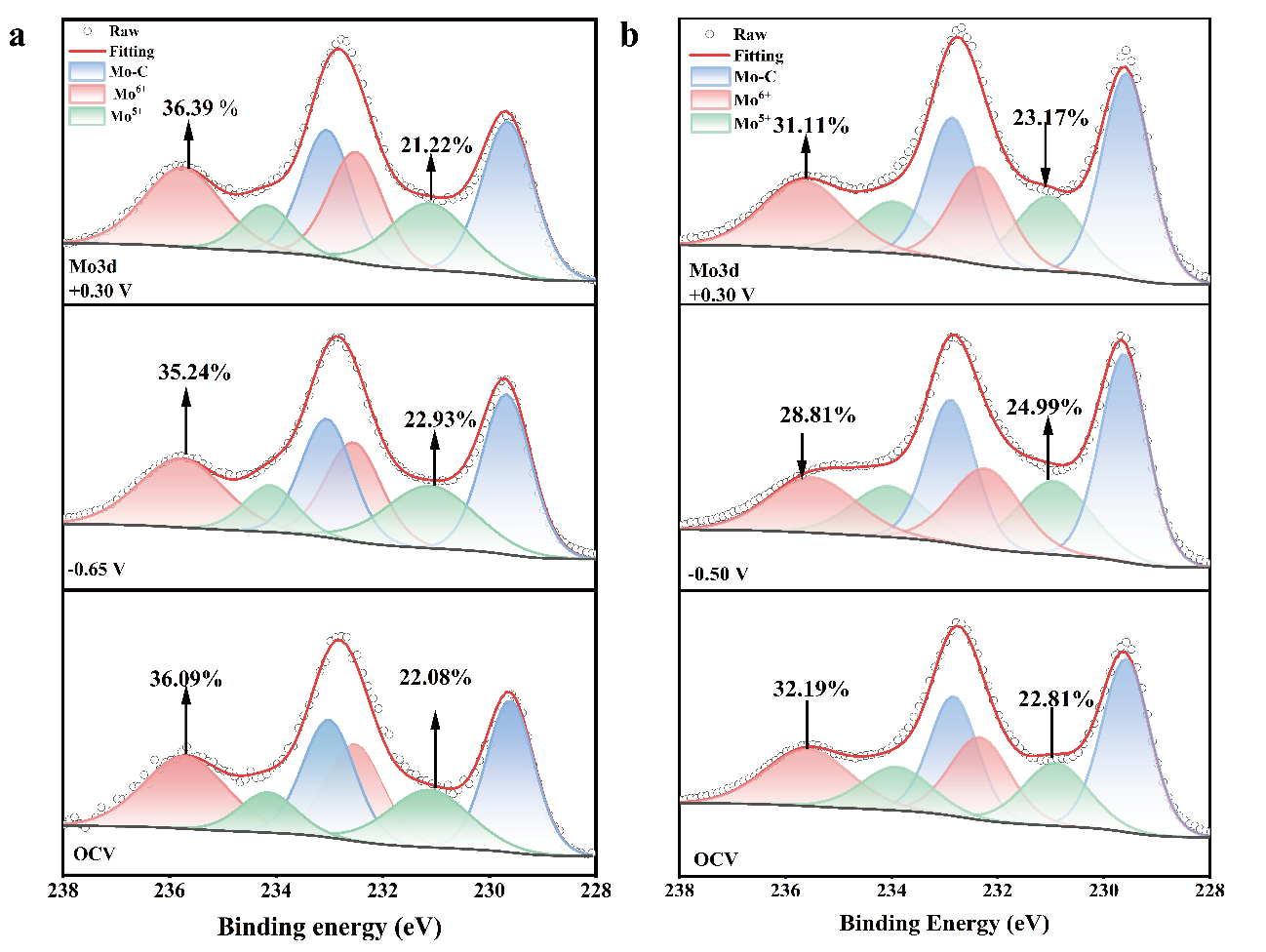


**Figure S10.** High-resolution XPS spectra of Mo 3d for the M:P = 5:1 composite in three electrochemical states, measured in (a) 1 M (NH_4_)_2_SO_4_ and (b) 0.1 M H_2_SO_4_.


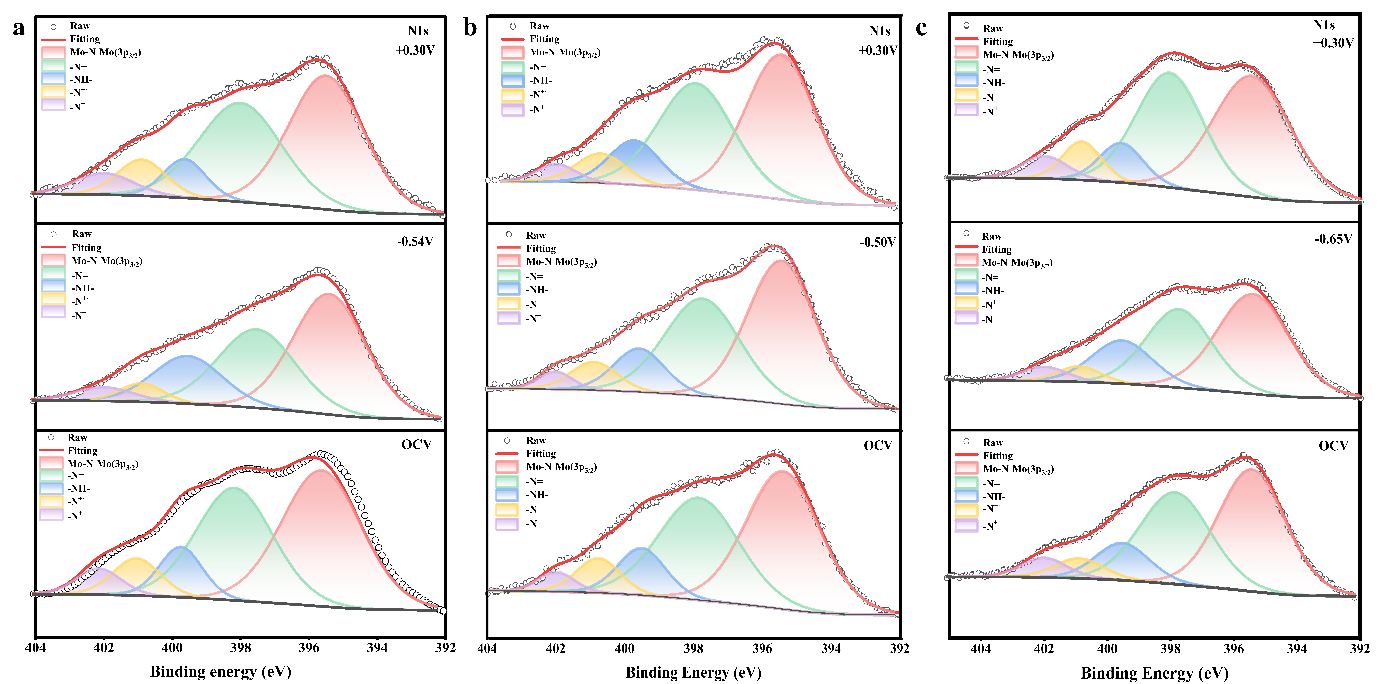


**Figure S11.** High-resolution XPS spectra of N 1s for the M:P = 5:1 composite in three electrochemical states, measured in (a) 1 M-0.1 M mixed electrolyte, (b) 0.1 M H_2_SO_4_, and (c) 1 M (NH_4_)_2_SO_4_.
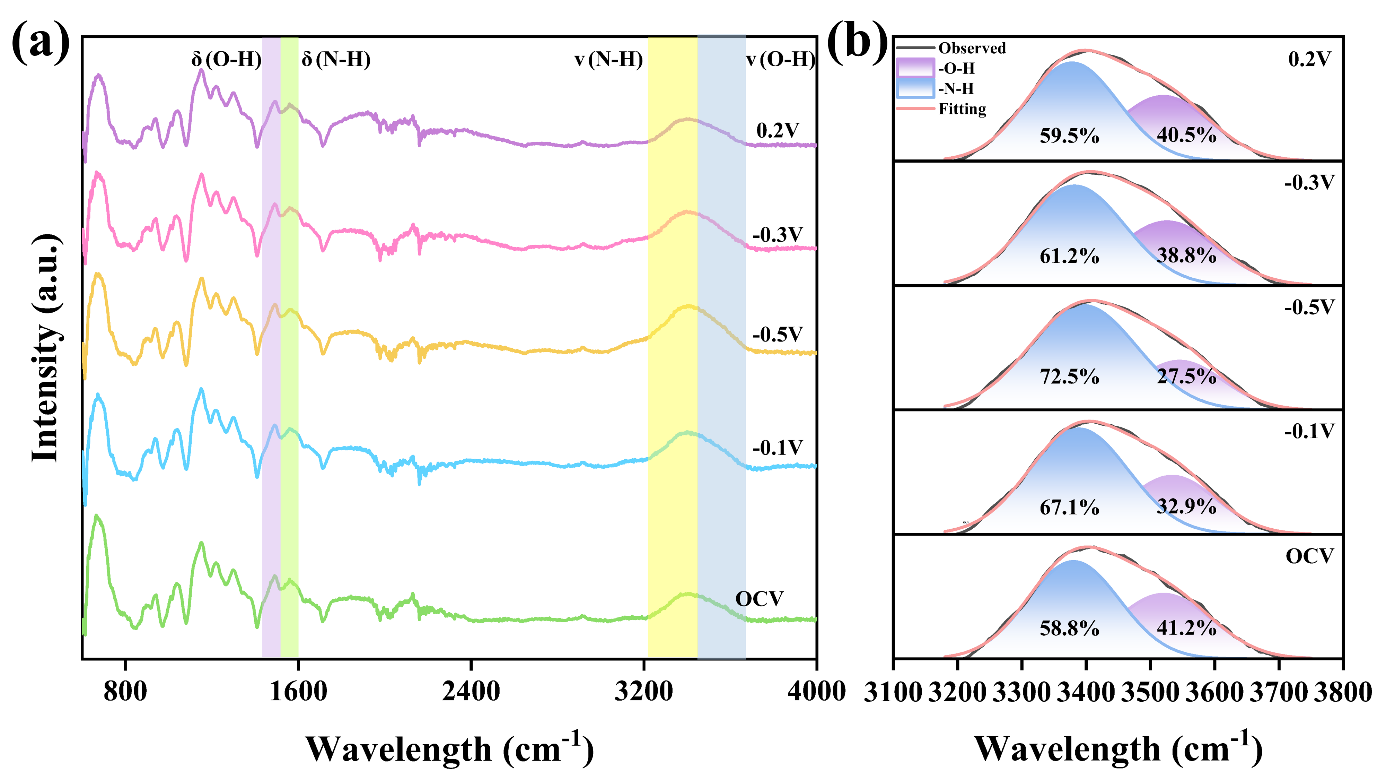
**Figure S12.** (a) In situ infrared spectrum of M:P = 5:1 composite in 1 M-0.1 M mixed electrolyte at different charge/discharge states. (b) Magnified image of 3100-3800 cm^-1^ wavelength.


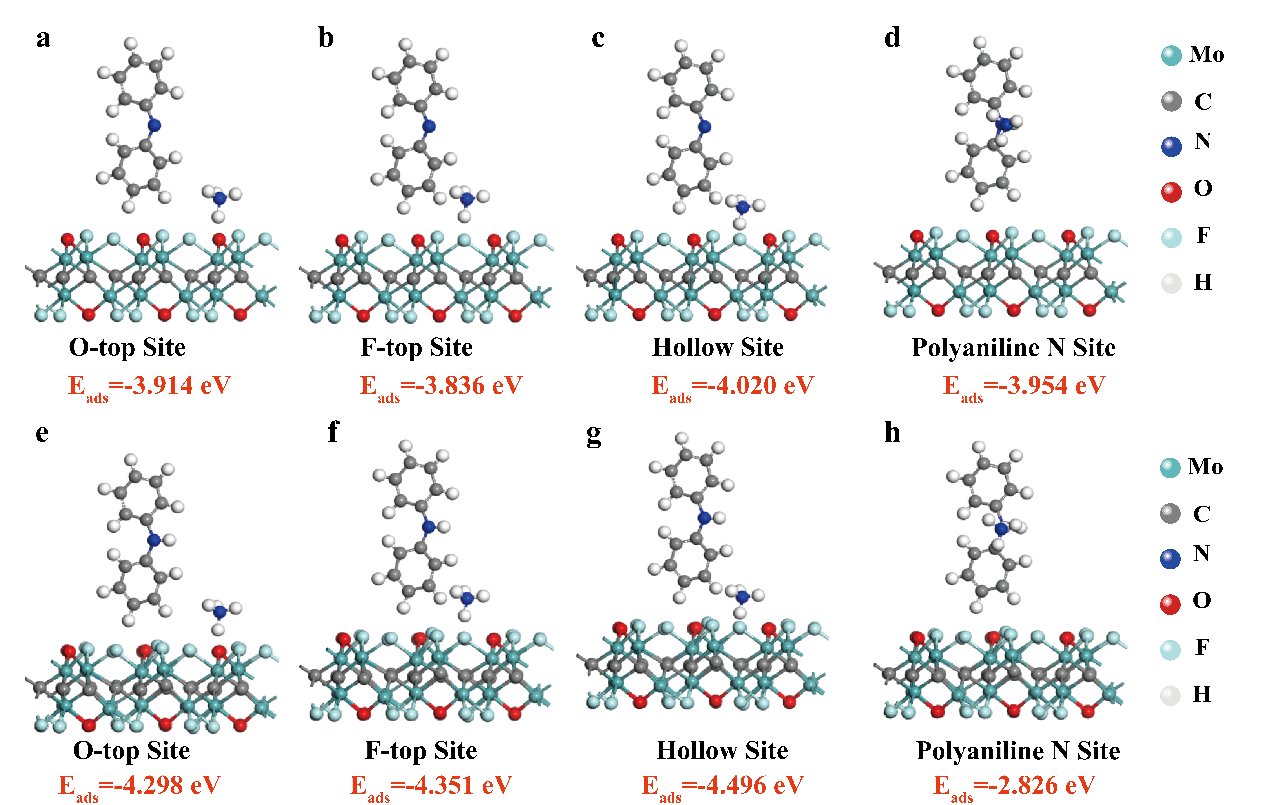
**Figure S13.** Optimized top-view configurations and corresponding NH_4_^+^ adsorption energies at different adsorption sites: (a-d) in 1 M (NH_4_)_2_SO_4_ and (e-h) in the 1 M-0.1 M mixed electrolyte.


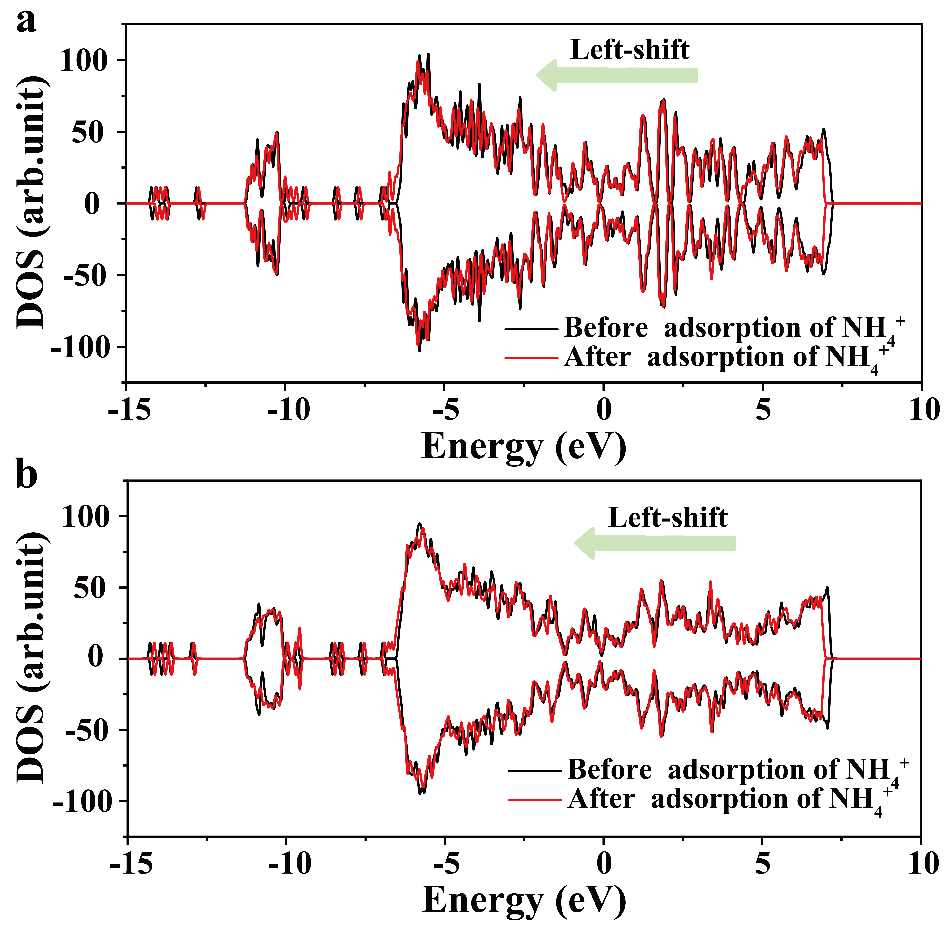


**Figure S14.** Total density of states (DOS) of the M:P = 5:1 composite before and after NH_4_^+^ adsorption for (a) the +5 valence state and (b) the +6 valence state.


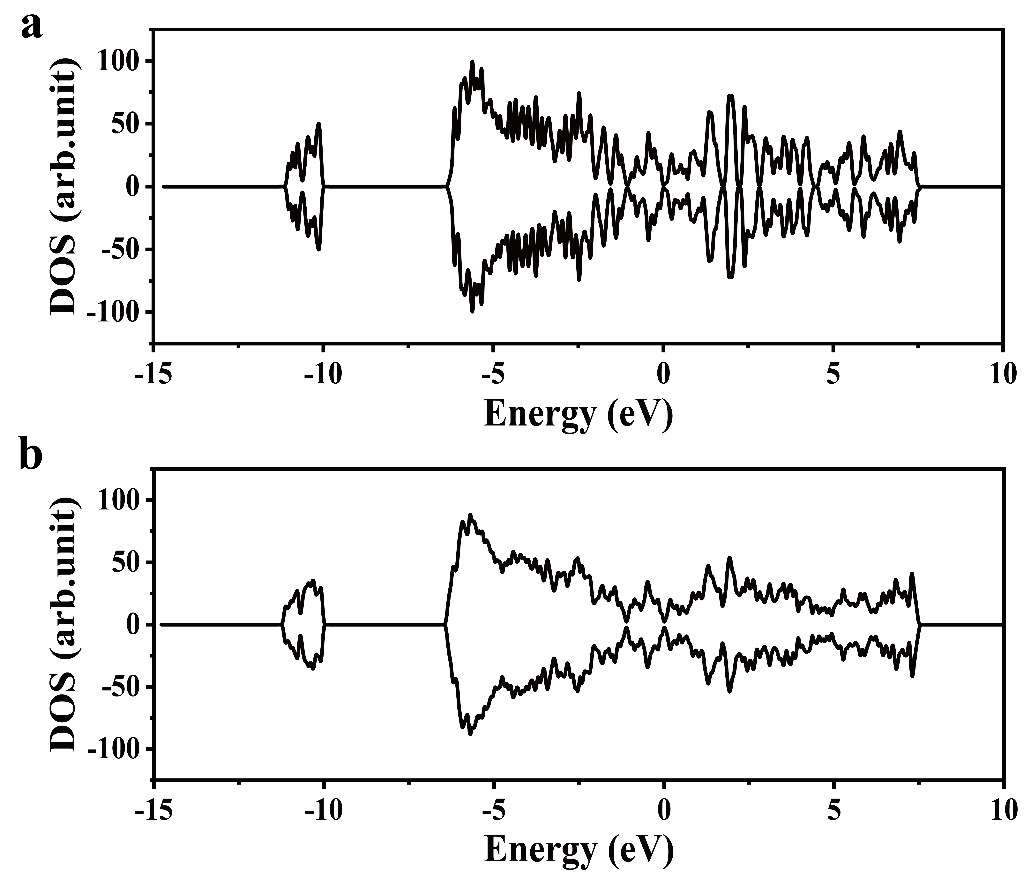


**Figure S15.** Total density of states (DOS) of the M:P = 5:1 composite in (a) the +5 valence state and (b) the +6 valence state.


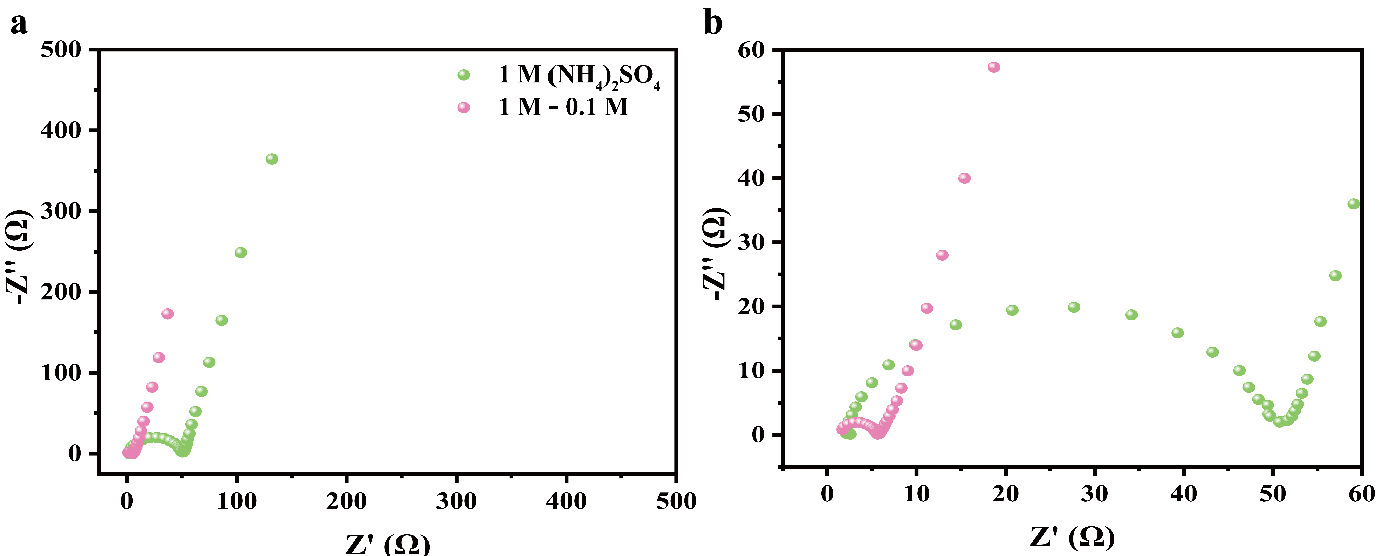


**Figure S16.** Electrochemical impedance spectrum and enlarged view of the high-frequency region for the M:P = 5:1 electrode in 1 M (NH_4_)_2_SO_4_ and 1 M-0.1 M mixed electrolytes.


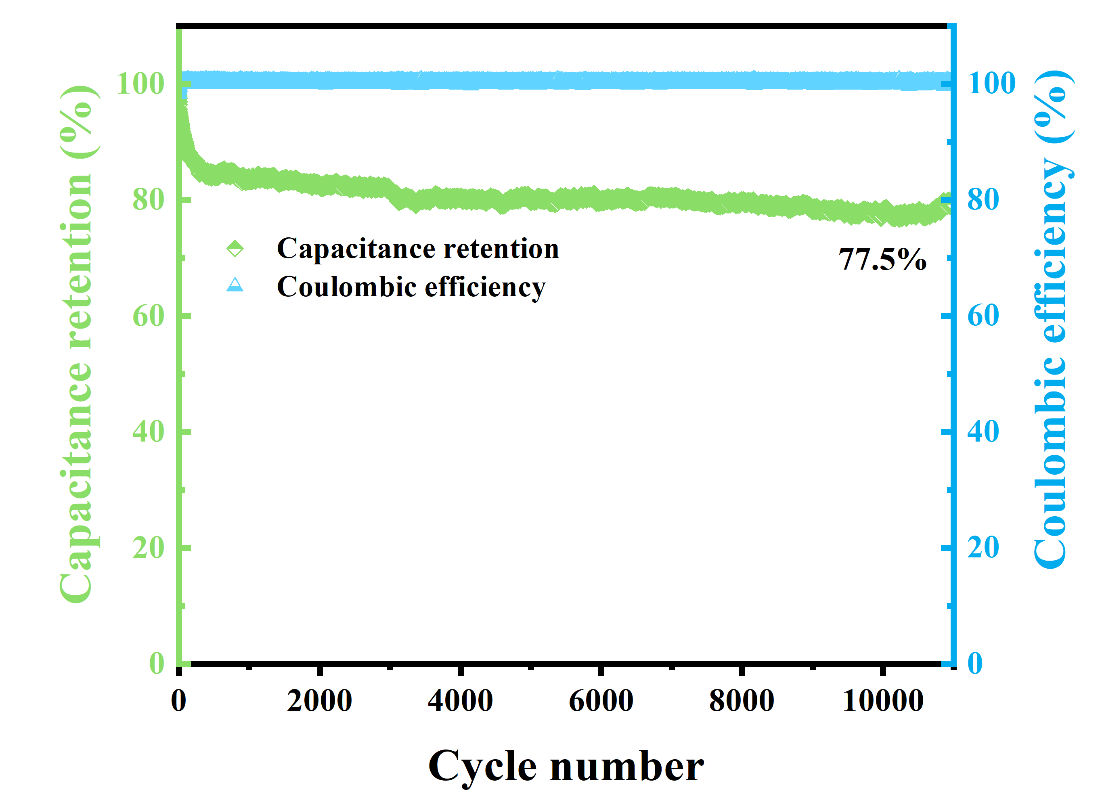


**Figure S17.** Long-term cycling performance of the composite at 1 A g^-1^ in 1 M (NH_4_)_2_SO_4_.


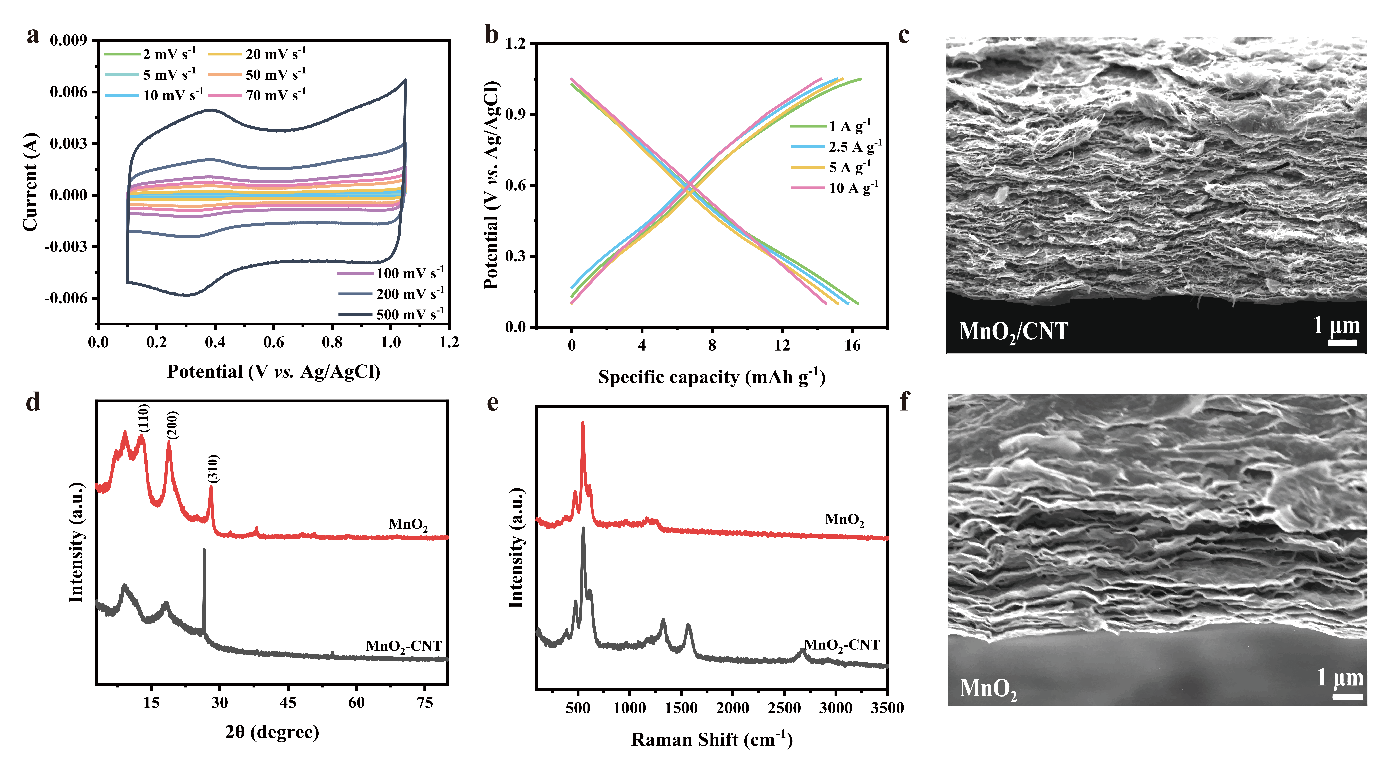


**Figure S18.** (a) CV curves of MnO_2_/CNTs at different scan rates. (b) GCD curves under different current densities. (c) SEM diagram of MnO_2_/CNTs. (d) and (e) XRD and Raman spectra of MnO_2_/CNTs and MnO_2_. (f) SEM diagram of MnO_2_.


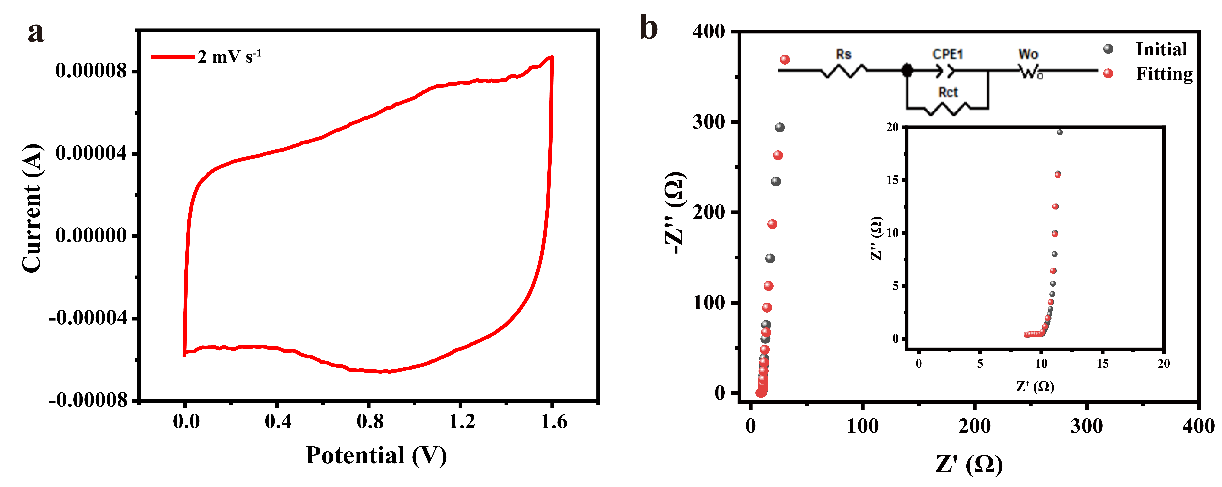


**Figure S19.** The performance of MnO_2_/CNTs||Mo_4/3_CT_z_/PANI cell. (a) CV curves at 2.0 mV s^-1^, (b) the Nyquist plot and equivalent circuits.

**Table S1.** Resistance parameters fitted from equivalent circuit models for PANI, Mo_4/3_CT_z_, and Mo_4/3_CT_z_/PANI composite films with varying ratios, based on EIS data.

| Resistance [Ω] | R_s_  [Ω] | R_ct_  [Ω] |
| --- | --- | --- |
| PANI | 2.14 | 66.91 |
| Mo_4/3_CT_z_ | 1.76 | 48.67 |
| M:P = 10:1 | 1.45 | 50.42 |
| M:P = 5:1 | 13.19 | 48.17 |
| M:P = 2:1 | 2.2 | 52.55 |
| M:P = 1:1 | 2.04 | 52.2 |

**Table S2.** Resistance parameters fitted using an equivalent circuit model for the M:P = 5:1 electrode in different electrolytes, based on EIS data.

| Resistance  [Ω] | R_s_  [Ω] | R_ct_  [Ω] |
| --- | --- | --- |
| 1 M (NH_4_)_2_SO_4_ | 13.19 | 48.17 |
| 0.1 M H_2_SO_4_ | 6.15 | 4.54 |
| 1 M-0.01 M | 11.73 | 16.87 |
| 1 M-0.1 M | 1.72 | 3.95 |
| 1 M-1 M | 31.74 | 12.96 |

**Table S3.** Mo 3d XPS peak assignments and corresponding atomic percentages for the M:P = 5:1 composite in different electrolytes.

| electrolyte | state |  | Mo-C | Mo3d_3/2_  (5+) | Mo3d_3/2_  (6+) | Mo-C | Mo3d_5/2_  (5+) | Mo3d_5/2_  (6+) |
| --- | --- | --- | --- | --- | --- | --- | --- | --- |
| 1 M-0.1 M | 0.30V | BE [eV]_n_ | 229.52 | 230.89 | 232.25 | 232.79 | 233.94 | 235.60 |
|  |  | Atomic Ratio (%) | 23.28 | 8.34 | 13.41 | 25.46 | 10.22 | 19.29 |
|  | -0.54V | BE [eV]_n_ | 229.67 | 231 15 | 232.35 | 232.94 | 234.20 | 235.70 |
|  |  | Atomic Ratio (%) | 24.60 | 12.00 | 9.04 | 26.52 | 13.30 | 14.53 |
|  | OCV | BE [eV]_n_ | 229.50 | 231.00 | 232.41 | 232.79 | 234.05 | 235.64 |
|  |  | Atomic Ratio (%) | 20.92 | 11.02 | 13.71 | 24.93 | 10.67 | 18.74 |
| 1M (NH_4_)_2_SO_4_ | OCV | BE [eV]_n_ | 229.61 | 231.15 | 232.52 | 233.01 | 234.15 | 235.71 |
|  |  | Atomic Ratio (%) | 17.59 | 11.99 | 17.72 | 23.04 | 10.09 | 19.58 |
|  | -0.65V | BE [eV]_n_ | 229.68 | 231.11 | 232.55 | 233.05 | 234.10 | 235.78 |
|  |  | Atomic Ratio (%) | 19.73 | 13.95 | 13.85 | 22.10 | 8.98 | 21.39 |
|  | 0.30V | BE [eV]_n_ | 229.66 | 231.13 | 232.50 | 233.05 | 234.19 | 235.75 |
|  |  | Atomic Ratio (%) | 19.70 | 12.72 | 14.74 | 22.60 | 8.59 | 21.65 |
| 0.1 M H_2_SO_4_ | OCV | BE [eV]_n_ | 229.60 | 230.92 | 232.35 | 232.85 | 233.92 | 235.59 |
|  |  | Atomic Ratio (%) | 22.67 | 11.04 | 13.32 | 22.34 | 11.77 | 18.87 |
|  | -0.50V | BE [eV]_n_ | 229.64 | 230.95 | 232.25 | 232.90 | 234.07 | 235.50 |
|  |  | Atomic Ratio (%) | 22.87 | 12.48 | 12.79 | 23.33 | 12.51 | 16.02 |
|  | 0.30V | BE [eV]_n_ | 229.49 | 230.85 | 232.30 | 232.75 | 233.90 | 235.54 |
|  |  | Atomic Ratio (%) | 22.95 | 10.17 | 14.35 | 23.00 | 10.19 | 19.33 |

**Table S4.** Comparison of representative full AIBs.

| Materials | Potential window | | electrolyte | capacity | stability | Energy Density | Ref |
| --- | --- | --- | --- | --- | --- | --- | --- |
| MnO_2_-CNTs\|\|  PTCDI-Ti_3_C_2_T_x_ MXene | 0-2.1V | PVA-NH_4_Cl hydrogel electrolyte | | 87.18 μAh cm⁻²  (1.0 mA·cm⁻²) | 81.67%  3000C | 82.48  μWh·cm^-2^ | [1] |
| MnO_2_-CNTs\|\|PTCDI-Ti_3_C_2_T_x_ MXene | 0-1.9 V | PAM gel electrolyte | | 87.69  mAh cm^-2^  (1.0 mA·cm⁻²) | 90.43%  2500C | 66.05  μWh·cm^-2^ | [2] |
| d-V_2_CT_x_\|\|Na_0.6_MnO_2_ | 0-1.6 V | 0.5 M NH_4_Ac | | 115.9 mAh g^-1^  (1.0 A g^-1^) | 58.2%  500C | 103.36  Wh kg^-1^ | [3] |
| NH_4_V_4_O_10_\|\|MoS_2_/MXene | 0-1.2 V | 0.5 M NH_4_Cl | | 58.5 mA h g^−1^  (50 mA g^−1^) | 83%  50C | - | [4] |
| MnO_2_/CNTs\|\|Mo_4/3_CT_z_/  PANI | 0-1.6 V | 1 M (NH_4_)_2_SO_4_  + 0.1 M H_2_SO_4_ | | 101.4 mAh g^-1^  (1.0 A g^-1^) | 75.7%  10,000C | 81.6  Wh kg^-1^ | **This work** |

References

[1] G. Kresse, J. Furthmüller, Efficient iterative schemes for ab initio total-energy calculations using a plane-wave basis set. *Phys. Rev. B* **1996**, *54*, 11169.

[2] G. Kresse, J. Furthmüller, Efficiency of ab-initio total energy calculations for metals and semiconductors using a plane-wave basis set. *Comput. Mater. Sci* **1996**, *6*, 15.

[3] P. E. Blöchl, Projector augmented-wave method. *Phys. Rev. B* **1994**, *50*, 17953.

[4] J. P. Perdew, K. Burke, M. Ernzerhof, Generalized Gradient Approximation Made Simple. *Phys. Rev. Lett.* **1997**, *77*, 3865.

[5] S. Grimme, J. Antony, S. Ehrlich, H. Krieg, A consistent and accurate ab initio parametrization of density functional dispersion correction (DFT-D) for the 94 elements H-Pu. *J. Chem. Phys*. **2010**, *132*, 154104.

[6] S. Grimme, S. Ehrlich, L. Goerigk, Effect of the damping function in dispersion corrected density functional theory. *J. Comput. Chem.* **2011**, *32*, 1456.
